# Considerations and practical implications of performing a phenotypic CRISPR/Cas survival screen

**DOI:** 10.1371/journal.pone.0263262

**Published:** 2022-02-17

**Authors:** Ator Ashoti, Francesco Limone, Melissa van Kranenburg, Anna Alemany, Mirna Baak, Judith Vivié, Frederica Piccioni, Pascale F. Dijkers, Menno Creyghton, Kevin Eggan, Niels Geijsen

**Affiliations:** 1 Hubrecht Institute, Developmental Biology and Stem Cell Research, Utrecht, The Netherlands; 2 Department of Stem Cell and Regenerative Biology, Harvard University Cambridge, MA, United States of America; 3 Stanley Center for Psychiatric Research, Broad Institute of MIT and Harvard, Cambridge, MA, United States of America; 4 Single Cell Discoveries, Utrecht, The Netherlands; 5 Broad Institute, Cambridge, MA, United States of America; Hirosaki University Graduate School of Medicine, JAPAN

## Abstract

Genome-wide screens that have viability as a readout have been instrumental to identify essential genes. The development of gene knockout screens with the use of CRISPR-Cas has provided a more sensitive method to identify these genes. Here, we performed an exhaustive genome-wide CRISPR/Cas9 phenotypic rescue screen to identify modulators of cytotoxicity induced by the pioneer transcription factor, DUX4. Misexpression of DUX4 due to a failure in epigenetic repressive mechanisms underlies facioscapulohumeral muscular dystrophy (FHSD), a complex muscle disorder that thus far remains untreatable. As the name implies, FSHD generally starts in the muscles of the face and shoulder girdle. Our CRISPR/Cas9 screen revealed no key effectors other than DUX4 itself that could modulate DUX4 cytotoxicity, suggesting that treatment efforts in FSHD should be directed towards direct modulation of DUX4 itself. Our screen did however reveal some rare and unexpected genomic events, that had an important impact on the interpretation of our data. Our findings may provide important considerations for planning future CRISPR/Cas9 phenotypic survival screens.

## Introduction

CRISPR/Cas9, which is now a highly popular and widely used genome editing technique, was initially discovered as the adaptive immune system of bacteria, to protect against viral infection [1, 2]. Although not the first genome editing method, CRISPR/Cas9 has proven to be much more user friendly due to its easy adaptability, and being more cost-, labor- and time-efficient compared to its predecessors: transcription activator-like effector nucleases (TALENs) [3–6] and ZINC-fingers nucleases (ZFNs) [7–11]. Its ability to knock out any gene by creating a double-stranded break [12–14] in such an easy manner, makes this technique very suitable for genome-wide loss-of-function studies. The advantages and ease of use of the CRISPR/Cas9 technology inspired us to perform a genome-wide screen on a FSHD *in vitro* model, to find potential modulators that contribute or aggravate the FSHD pathophysiology. Successful performance of a FSHD genome-wide screen will critically depend on the cell system being used. The cells should be highly proliferative, easily transfected and display a robust DUX4-induced phenotype.

Facioscapulohumeral muscular dystrophy (FSHD) is an autosomal dominant degenerative muscle disease. It is one of the most prevalent neuromuscular disorders [15], characterized by progressive and asymmetric muscle weakness which generally starts in facial muscles, and then slowly progresses to muscles of the shoulders, upper limbs and eventually the lower extremities [16]. The age of onset is highly variable, but calculations based on a 122-case study demonstrate that the mean age of onset is in the early twenties [17]. The primary cause of the disease is the misexpression of the double homeobox 4 (DUX4) transcription factor, due to failure in epigenetic silencing [18–20]. DUX4 is normally expressed early in development in the cleavage stage embryo [21, 22], in the adult testis [20] and in the thymus [23]. De-repression of DUX4 in muscle activates a large cascade of events, triggering the activation of many pathways [22, 24–33], with target genes being involved in biological processes such as RNA splicing and processing (DBR1 [24, 34–36], CWC15 [24, 34, 36], PNN [24, 35], CLP1 [24, 35, 36], TFIP11 [24, 34–36]), spermatogenesis (CCNA1 [24, 34–36], ZNF296 [24, 34–36], TESK2 [24, 34, 35]), early embryonic development (ZSCAN4 [24, 34–36], LEUTX [34–36], STIL [24, 34, 35]), protein processing and degradation (SIAH1 [24, 34–36], RHOBTB1 [24, 34, 35], TRIM36 [24, 34, 35]), and cell motility and migration (CXCR4 [24, 34, 35], ROCK1 [24, 35], SNAI1 [24, 34–36]).

We hypothesized that of one or more factors downstream of DUX4 expression are responsible for the rapid apoptotic response that follows DUX4 induction. Knowing if there are key downstream targets of DUX4 can have important clinical applications as they could direct intelligent therapeutic design. We tested this hypothesis by performing a genome-wide CRISPR/Cas9 knockout screen.

DUX4 is a so-called pioneer factor [37, 38], capable of regulating its target genes independent of their chromatin state, in the human context. The network of genes activated by pioneer factors is therefore less affected by cellular identity. Indeed, Jones and colleagues have demonstrated that DUX4 activates the same downstream target genes in human B-lymphocytes as previously identified in human skeletal muscle myoblasts [39, 40]. Using an adherent leukemic cell line that is frequently used for genome-wide screening purposes (KBM7 [41, 42]), we performed an exhaustive CRISPR knockout screen to identify factors that could mitigate DUX4-induced cytotoxicity. We inserted a doxycycline-inducible DUX4 transgene into the KBM7 cells [41, 42] to generate DUX4 inducible expression (DIE) cells. Using the Brunello CRISPR/Cas9 library [43], we screened for modulators of DUX4 cytotoxicity. Our results suggest that no single gene knockout, other than DUX4, is capable of rescuing DUX4-triggered apoptosis in our transgene model system.

This study does however, provide interesting insights into critical parameters that need to be considered when executing a genome-wide CRISPR screen.

## Results

### Generation and validation of a DUX4-inducible cell line

To perform a successful genome-wide screen, a cellular model is required that is highly proliferative, has a high viral transduction efficiency and displays a robust phenotype. An adherent clone of the KBM7 cell line possess all these characteristics and has already been used extensively in a wide variety of functional screens [42, 44–52]. The cells were initially reported to be near-haploid [41, 42], but subsequently rediploidized [53, 54]. These adherent diploid KBM7 cells were used for the generation of our inducible DUX4 cell line.

**Fig 1:**
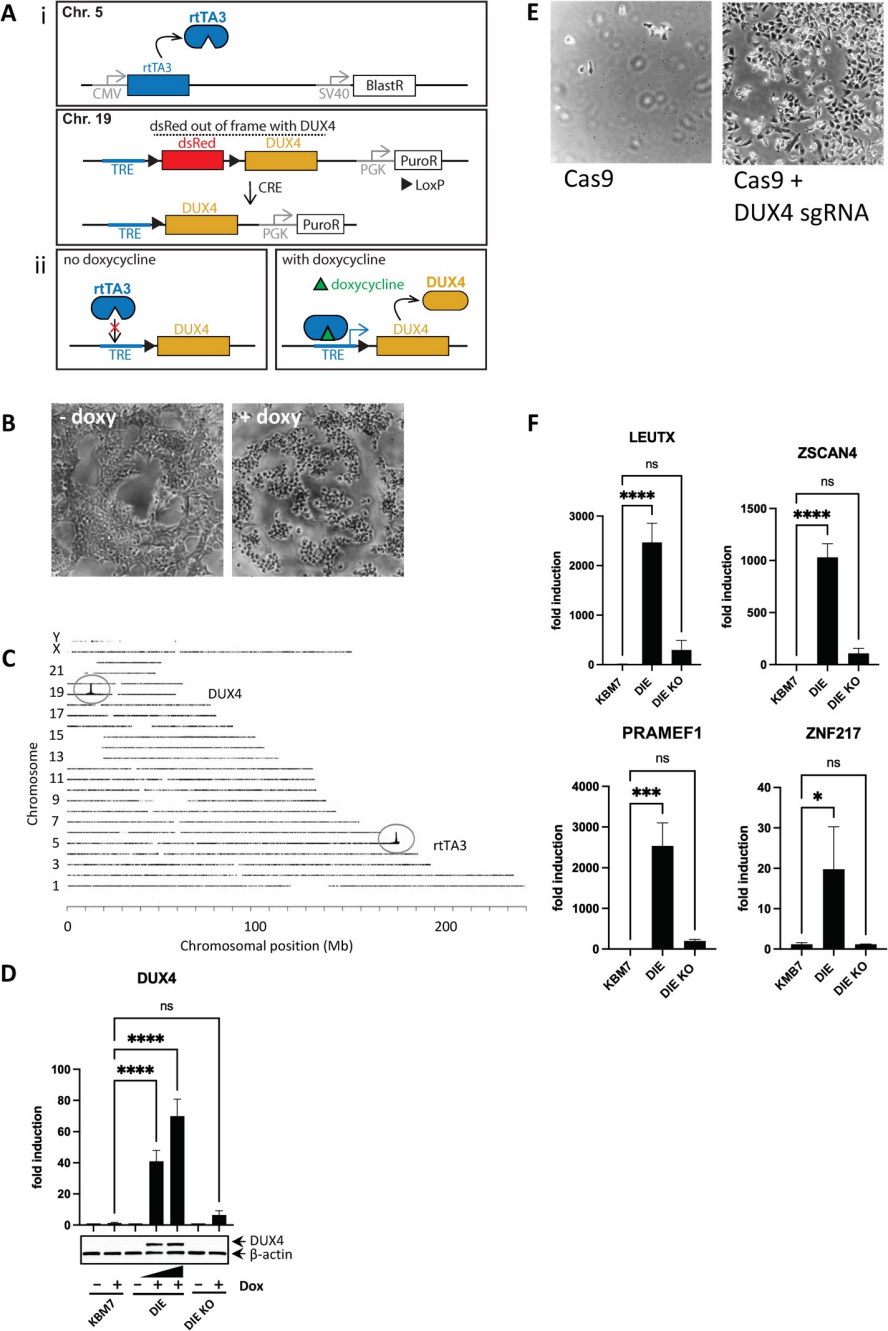
Creation and validation of the DIE cell line. **(A)** Constructs expressed in the DIE cell line to induce expression of DUX4. i) Top: Schematic representation of the rtTA3 construct (integrated on chromosome 5), constitutively expressing rtTA3 (under control of the CMV promoter) with selection marker blasticidin (BlastR, SV40 promoter). Bottom: LSL (LoxP-dsRed-stop-LoxP)-DUX4 cassette under control of TRE, tetracycline-responsive element, inducible by tetracycline-derivative doxycycline, together with selection marker puromycin (PuroR, PGK promoter), integrated on chromosome 19. dsRed but not DUX4 can be induced, as DUX4 is out of frame with dsRed. Action of CRE recombinase removed dsRed between the LoxP sites, enabling doxycycline-inducible DUX4 expression. ii) In the absence of doxycycline, rtTA3 does not bind to the TRE; addition of doxycycline results in rtTA3-TRE binding and subsequent transcription of DUX4. **(B)** Phase-contrast images of DIE cells without doxycycline and 24h after doxycycline exposure (1000ng/ml). **(C)** Schematic representation of transgene integration sites within human genome, by TLA analysis. The inducible DUX4 cassette maps to the p-arm of chromosome 19, and the rtTA3 transgene maps to the end of the q-arm of chromosome 5. **(D)** Analysis of DUX4 expression by qRT-PCR and western blot in KBM7, DIE and DIE-KO cells (DIE cells with knockout for DUX4) with or without doxycycline addition (500ng/ml and 1000ng/ml in DIE cells, 1000ng/ml in KBM7 and DIE KO cells), as detected by qRT-PCR (top panel) and western blot analysis (bottom panel). β -actin was used as a loading. Fold induction was calculated by 2^-(ddCT) of untreated or doxycycline-treated cells normalized by HPRT expression. Statistical significance was determined by ANOVA analysis. **(E)** Phase-contrast images of DIE cells which were transduced with either Cas9 protein, or Cas9 protein with DUX4 sgRNA prior to treatment with doxycycline (1000ng/ml) for at least 24h. Dead cells were removed by a DPBS wash to expose the surviving population. **(F)** Induction of mRNA expression of known downstream targets of DUX4 upon doxycycline treatment (1000ng/ml) in KBM7, DIE and DIE-KO cells, as measured by qRT-PCR. Fold induction was calculated by 2^-(ddCT) of uninduced or doxycycline-induced cells normalized by HPRT expression. Statistical significance was determined by ANOVA analysis.

Low levels of DUX4 expression can efficiently induce apoptosis [20, 34, 55], which interfered with the generation of our model system. To circumvent premature DUX4 toxicity caused by leaky expression of the Tet-On system [56, 57], we inserted a LoxP-DsRed-LoxP stop-cassette (LSL) in between the Tet-operator and a DUX4 transgene. The DUX4 transgene element itself consisted of the three exons (starting with the translational start site) and the two introns of the DUX4 gene, including the polyA sequence. This is the same sequence found in the most common pathology-associated haplotype, 4A161 [58]. This construct was introduced in the adherent KBM7 cells in combination with a constitutive rtTA3-expressing construct. After stable integration of the construct, the stop-cassette was removed using CRE recombinase, placing *DUX4* under the control of the TRE, that can be induced by rtTA3 (Fig 1A). Eighty monoclonal lines were derived by single-cell flow cytometry sorting, of which one displayed tight doxycycline-dependent DUX4 induction and robust cell death upon doxycycline addition (Fig 1B and S2A Fig). A monoclonal cell line was derived from this positive clone, which we named the ‘DUX4-Inducible Expression’ (DIE) cell line.

To further analyze the functional effect of DUX4 induction, DIE cells were stained with AnnexinV-Alexa Fluor 488 and propidium iodine (PI) (S2B Fig). As shown in the supplementary videos (S1-S3 videos), during 12 hours of doxycycline exposure DIE cells became positive for the apoptotic marker AnnexinV. To show that the apoptotic phenotype was dependent on the induction of the DUX4 transgene, we targeted the DUX4 transgene using CRISPR/Cas9. To target the DUX4 transgene, 2 independent single guide RNAs (sgRNAs) were used, one targeting the C-terminal domain of the DUX4 open reading frame (ORF) and the other close to the polyA tail of DUX4, thus generating DUX4 knockout DIE cells (DIE-KO). RT-qPCR and Western blot (WB) analysis of the DIE and the DIE DUX4 knockout (DIE KO) cells demonstrated successful knockout of the DUX4 transgene at both RNA and protein level (Fig 1D). CRISPR/Cas9-mediated targeting of the DUX4 transgene successfully rescued the DIE cells from apoptosis upon doxycycline administration (Fig 1E), demonstrating that apoptosis upon doxycycline administration in the DIE line is mediated by DUX4. DUX4 induction in the DIE cells resulted in induction of its known downstream target genes (LEUTX, ZSCAN4, PRAMEF1 and ZNF217) but not in DIE KO cells (Fig 1F), demonstrating that inducing DUX4 expression induces downstream target genes that are also induced by endogenous DUX4.

### DUX4 gene expression signature in DIE cells

**Fig 2:**
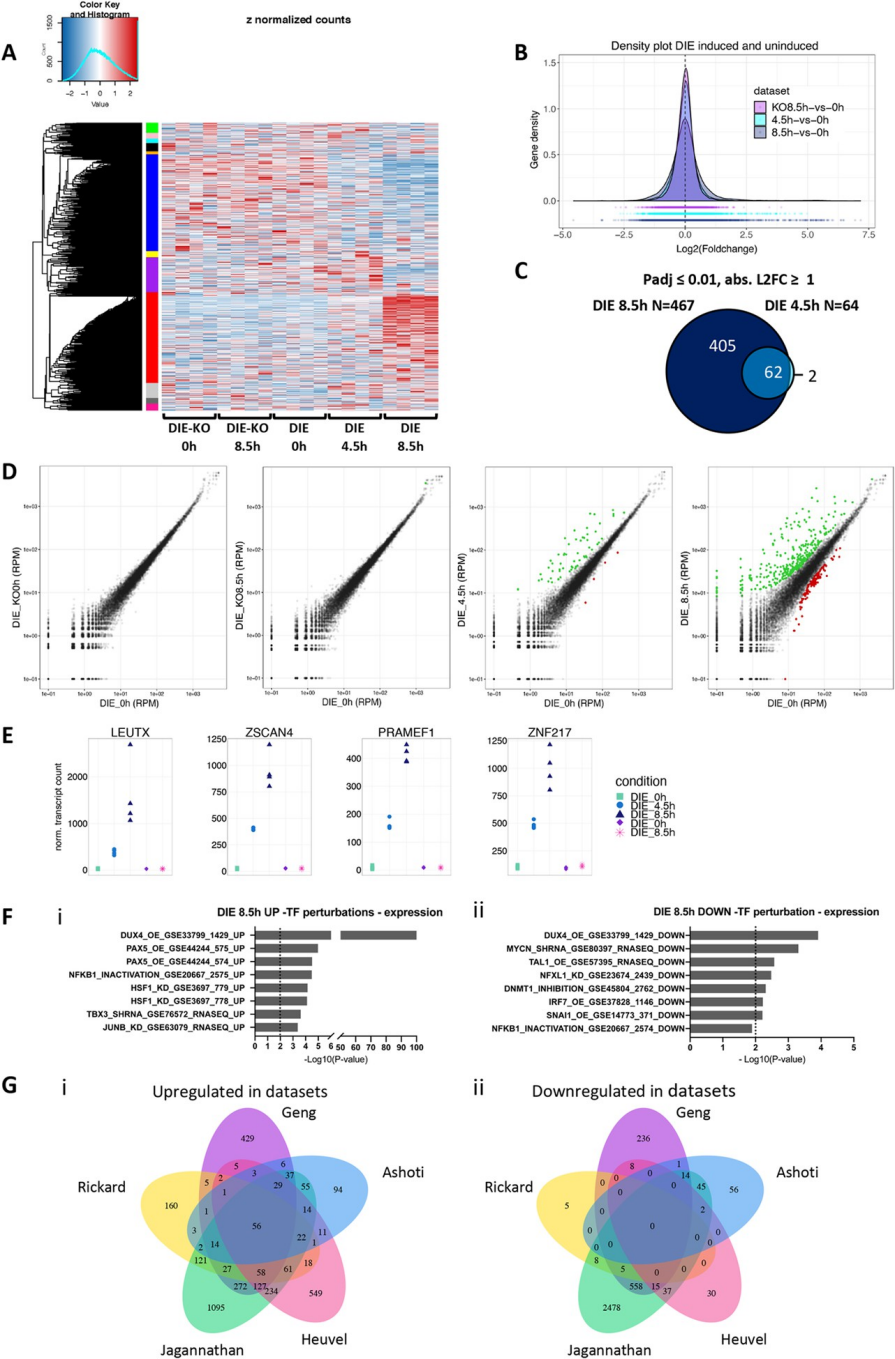
RNA-sequencing data reveals differentially expressed genes upon DUX4 expression. **(A)** Heatmap showing differentially expressed genes between DIE cells and DIE-KO (knockout for DUX4) cells, untreated or treated with doxycycline (1000ng/ml) for 4.5h or 8.5h, with gene clusters (color-coded) on the y-axis, and samples on the x-axis. **(B)** Gene density plot demonstrating the effects of DUX4 expression on the transcriptome of the DIE cell line. Doxycycline addition (1000ng/ml) for 4.5h or 8.5h results in an increase of differentially expressed genes compared to uninduced DIE cells, as indicated by the bell shape widening and shortening. This effect was not seen doxycycline-treated DIE-KO cells. **(C)** Venn diagram showing the overlap and the number of differentially expressed genes after 4.5h and 8.5h of doxycycline addition (1000ng/ml) (adjusted P-value ≤ 0.01, and absolute Log2FC ≥ 1). **(D)** Scatter plots of gene expression (RPM: Reads per million) of doxycycline-treated (1000ng/ml) DIE cells versus untreated DIE cells. The left two panels represent uninduced DIE cells (DIE_0h) on the x-axis versus doxycycline-treated or untreated DIE-KO samples (KO_0h and KO_8.5h) on the y-axis and show that addition of doxycycline has no effect on gene expression in DIE-KO cells. The right two panels compare the doxycycline-treated DIE cells with untreated DIE samples (4.5h and 8.5h). Green and red points represent the differentially expressed genes with an adjusted P-value ≤ 0.01, and absolute Log2FC ≥ 1. Green points represent upregulated genes, and the red points represent downregulated genes. **(E)** Count plots showing UMI and between sample normalized transcript counts of 4 known DUX4 targets genes: LEUTX, ZSCAN4, PRAMEF1 and ZNF217, in uninduced and doxycycline-treated (1000ng/ml) DIE cells and doxycycline-treated (1000ng/ml) DIE-KO cells. Every sample has 4 technical replicates, represented by 4 symbols. **(F)** Transcription factor perturbations analysis identifying transcription factors that are linked to the i) upregulation and ii) downregulation of the differentially expressed genes found in this study. Activation: OE or ACTIVATION, inhibition: KO, KD, SIRNA, SHRNA, INACTIVATION, or INHIBITION. **(G)** Quintuple Venn diagram comparing genes that are following DUX4 expression i) upregulated and ii) downregulated in this study (Ashoti) to those found in previous transcriptomic studies (Geng with P-value ≤ 0.01, FDR ≤ 0.05, abs L2FC ≥ 1; Rickard with Padj value of < 0.005 and abs L2FC > 2; Jagannathan with P-value ≤ 0.01, FDR ≤ 0.05, abs L2FC ≥ 1, Heuvel with P-value ≤ 0.005, FDR ≤ 0.05, abs L2FC ≥ 1).

Next, we analyzed the downstream transcriptional changes that were induced by DUX4 in the DIE cells by RNA sequencing. We compared 4 induced and uninduced technical replicates of two lines: the DIE line, and the DIE-KO line, where addition of doxycycline does not induce DUX4 expression (Fig 1D). As shown in Fig 2A, doxycycline treatment resulted in progressive temporal changes in gene expression in DIE cells, but not in DIE-KO cells, demonstrating DUX4-specific gene induction. Fig 2B shows the magnitude of the combined transcriptional changes induced by DUX4, visualized by comparing DIE cells to DIE-KO cells at different time intervals which emphasizes both the increasing number of differentially transcribed genes as well as the speed at which these transcriptional changes occur over time. Indeed, DUX4 induction resulted in a profound and progressive increase in the number of differentially expressed genes, with 64 differentially expressed transcripts at 4.5 hours post induction and 467 differentially expressed transcripts at 8.5 hours after induction (Fig 2B and 2C). The number of induced genes is greater than those with reduced expression levels, as can be seen in Fig 2B, consistent with DUX4’s role as a pioneer transcription factor [37]. Differential expression analysis reflects this, demonstrating more differentially upregulated genes in both induced DIE samples compared to uninduced DIE samples [Padj value ≤ 0.01, Log2fold change ≥ 1] (Fig 2B and 2D, S1 Table). Most differentially expressed genes are shared between the two induced samples that were treated with Doxycycline for 4.5 or 8.5 hours (Fig 2C). Among the upregulated genes are well known downstream targets of DUX4, including LEUTX, ZSCAN4, PRAMEF1 and ZNF217 (Fig 2E). We next used Enrichr [59, 60] to search for other similar studies that show similarities in their transcriptome. Based on Enrichr entries, the upregulated genes in induced DIE cells are linked to DUX4 activity [-Log10(P-value) = 100.3], as are the downregulated genes [-Log10(P-value) = 3.8] (Fig 2F and S2 Table). It shows high similarity between data from our study and one other DUX4 study that has been entered into the Enrichr database (GSE33799) [24]. Interestingly, PAX5 appears to also be linked to the upregulation of some of the same genes as DUX4 (Fig 2F). The PAX7 and PAX3 members of the Paired box (PAX) family of genes have previously been linked to FSHD and DUX4 [30, 61, 62]. While PAX5 itself may not be associated with FSHD, it binds to the same target sequence as PAX3, PAX7 and DUX4, which may explain how DUX4 overexpression might therefore induce similar downstream target genes as previously reported for PAX5.

We next compared the list of differentially expressed genes in DIE cells that were induced for 8.5h (DIE_8.5h) with 4 other studies that have previously explored the DUX4 transcriptional network in myoblast models or patient-derived muscle biopsies (Geng, Rickard, Jagannathan and Heuvel) [24, 34–36]. As shown in Fig 2G (panel i), we observed a high percentage of overlap between our dataset and those previously reported. The overlap between the upregulated genes is greater than the overlap that can be seen in the downregulated genes (Fig 2G, panel ii). This applies not only for the overlap seen between our data and the other datasets, but also between the 4 other datasets. This confirms that DUX4 can directly activate transcription, and that the downregulated genes might be more influenced by cell type. In addition, overlapping data seen here are likely an underrepresentation, due to the presence of gene families containing paralogs and pseudogenes in either reference genome databases, which can lead to multi-mapped or ambiguous reads [63]. To conclude, data shown here strongly suggest that in our DIE cell system, DUX4 induces transcriptional changes similar to those found in myoblasts from FSHD patients.

### Genome-wide CRISPR screen reveals large chromosomal truncations

**Fig 3:**
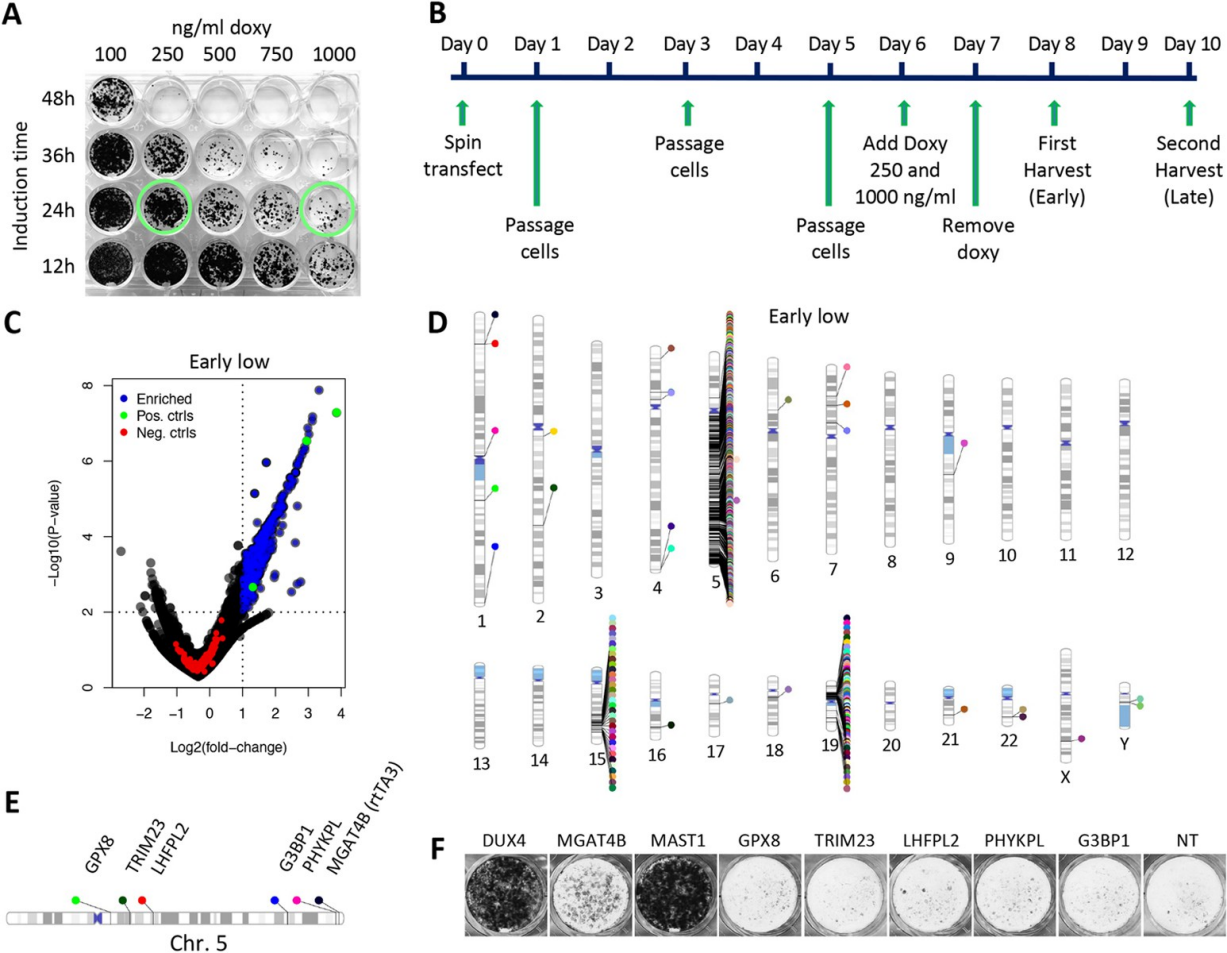
CRISPR Screen setup and discovery of a CRISPR/Cas9 screening artefact. **(A)** Viability staining of DIE cells treated with doxycycline (Doxy) concentrations (100, 250, 500 or 1000ng/ml) and for different exposure times (2, 4, 6, or 8h) to determine the optimal concentration and exposure time to induce sufficient cell death rates in DIE cells. Green circles indicate which conditions (low Doxy: 250ng/ml high Doxy: 1000ng/ml); were used for the genome-wide CRIPSR/Cas9 screen. **(B)** The CRISPR/Cas9 screen timeline from the time of library transfection (Day 0) to the final harvest of surviving DIE cells (Day 10). 6 days after transfection of the library, Doxycycline (Doxy) was added for 24h to induce DUX4 expression. Low Doxy: 250ng/ml; high Doxy: 1000ng/ml; early harvest: 24h after Doxy removal; late harvest: 48h after Doxy removal. **(C)** Volcano plot showing the enrichment of sets of guides of the low doxycycline 250ng/ml) -early harvest (24h after doxycycline removal) screen (early-low). Blue points represent guide sets that are significantly enriched (P-value ≤ 0.01), LFC ≥ 1), green points are the positive controls (DUX4, MAST1, MGAT4B), red points represent the non-target/negative control guides. **(D)** Chromosomal ideogram indicating the location of enriched hits in the human genome, of the low doxycycline-early harvest screen (see panel B). **(E)** Schematic representation of the location of a small number of false positive hits on chromosome 5 and chromosome 19. **(F)** Viability staining demonstrating surviving DIE-Cas9 cells (DIE cells constitutively expressing Cas9) after 250ng/ml doxycycline exposure, containing knockouts of the same genes mentioned in (E), but also DUX4, MGAT4B and MAST1. Media did not contain any selection markers (blasticidin or puromycin) to select for the presence of the rtTA3 or the DUX4 transgene. NT: Non-target controls.

Using our DIE cell system, we aimed to identify modulators of DUX4 cytotoxicity by performing a genome-wide CRISPR/Cas9 knockout screen. The Brunello human CRISPR knockout pooled library, optimized for maximal on-target activity and minimal off-target activity, was used for this purpose [43]. This library contains 77.441 sgRNAs, targeting all protein-coding genes, with an average of 4 sgRNAs per gene as well as 1000 non-coding control sgRNAs. To optimize the signal-to-noise ratio of the experimental system, we titrated the timing and dose of the doxycycline-mediated DUX4 induction (Fig 3A) and selected two doxycycline concentrations: low (250ng/ml) and high (1000ng/ml) doxycycline, with an exposure time of 24h). At these concentrations, respectively 95 to 99% of the cells die. Fig 3B outlines the setup of the screen. In addition to the high and low doxycycline concentrations, cells were harvested at two timepoints after doxycycline addition to allow recovery, early harvest (24h) and late harvest (72h), ultimately resulting in 4 separate 4 screens: low doxycycline/early harvest, low doxycycline/late harvest, high doxycycline/early harvest, and high doxycycline/late harvest.

Following doxycycline administration and induction of DUX4 expression, surviving cells were harvested, genomic DNA was extracted, and the sgRNA sequence was amplified and sent for sequencing. Sequencing results of the treated samples revealed many significantly enriched hits (Fig 3C and S3 Fig). These hits included DUX4 itself, demonstrating the sensitivity of the screen. In addition, we identified hits that rescued cells as well as the DUX4 sgRNAs did.

Upon closer examination, we saw that most of these enriched guides were located on the q arm of chromosome 5 (Fig 3D), suggesting that the q-arm in its entirety contains an essential modulator of cell survival following doxycycline addition. Indeed, the rtTA3 transgene responsible for DUX4 induction is located on the 5q arm (Fig 1C). This suggests that targeting Cas9 to the q arm of chromosome 5 resulted in the removal of the rtTA3 transgene, potentially through generation of a large deletion, a chromosomal truncation, or a chromosomal rearrangement. As the rtTA3 integration site is located at the end of chromosome 5q, single guide RNAs targeting genes upstream of this site (towards the centromere) can potentially cause a Cas9-mediated truncation, thereby removing the rtTA3 (Fig 3D, for phenograms of all 4 screens see S4 Fig). The correlation between the significance of a hit and its position along chromosome 5 highlights the strong association of these unexpected chromosomal rearrangements and the integration of rtTA3 at the end of chromosome 5, where the most significant hits reside in all four screens (S5 Fig). We also identified an enrichment of sgRNAs on chromosome 19 that enhanced survival at the location where the DUX4 transgene was inserted (Fig 1C). The effect of the sgRNAs that target Cas9 to this locus is possibly a result of interfering with expression of the DUX4 transgene, rather than interfering with expression of the genes that the sgRNAs are directed against.

The hotspot of hits on chromosome 15 can be explained by the genetic makeup of the original HAP1 cell line. In this cell line, a region of chromosome 15 integrated on chromosome 19q [64]. The hotspot of hits on chromosome 15 correlates to the region that has integrated on chromosome 19q, close to the MED25 site. Thus, targeting the region of chromosome 15 that has integrated on chromosome 19 possibly interferes with the DUX4 transgene expression, and at the same time also targets the same region in chromosome 15.

To confirm whether the identified hits act by inducing a knockout of their target gene or via an alternative mechanism, we tested some of these guides (the chromosomal locations of the genes they target are shown in Fig 3E) individually in DIE cells constitutively expressing Cas9 (DIE-Cas9 cells). In this experiment, we did not select for the rtTA3 or DUX4 transgene (Fig 1A), the reason for this is that the absence of selection for the transgene allows us to pick up deletions that involve the rtTA3 transgene (including the selection marker). No increased survival was detected compared to the background-surviving cells that were seen in the non-target control situation (Fig 3F). This suggests that the Cas9-induced truncation of a chromosomal arm and subsequent removal of rtTA3 activity is a rare event that was only identified due to the high sensitivity of our screen.

### Filtering the hits of the genome-wide CRISPR screen results revealed no single candidate

**Fig 4:**
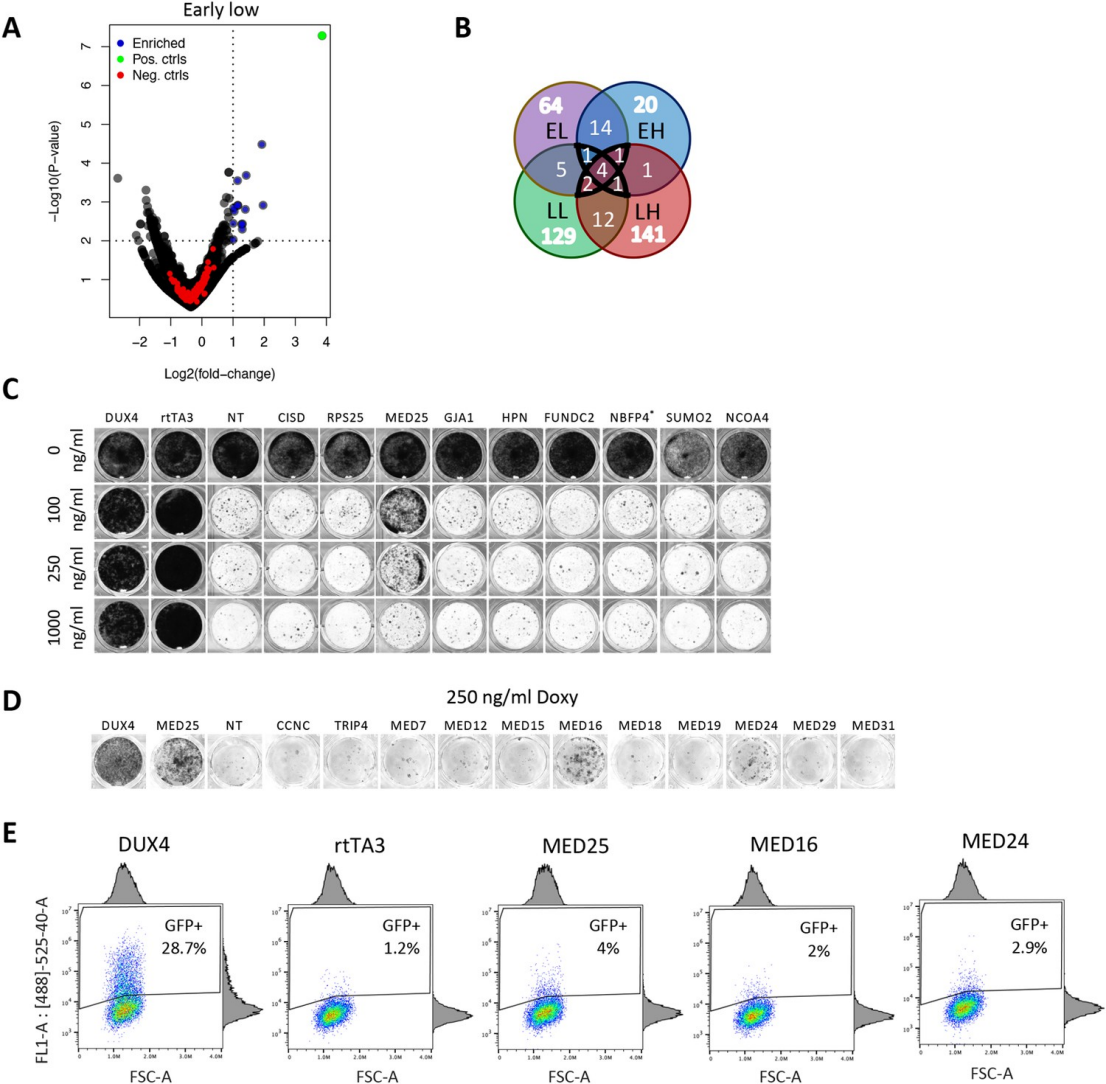
Filtered CRISPR screen data and validation of potential hits. **(A)** Adjusted volcano plot of screen data with low doxycycline (250ng/ml)/early harvest (24h after doxycycline removal, see Fig 3B) showing the enrichment of sets of guides targeting genes not located on chromosome 5q or chromosome 19p. Blue points represent guide sets that are significantly enriched (P-value ≤ 0.01), Log2(fold change) ≥ 1), the green point is the positive control (DUX4), red points represent the non-target control guides. **(B)** Venn diagram showing the overlap of filtered hits between the four screens (EL: Early harvest-low Doxy, LL: Late harvest-Low doxy, EH: Early harvest-high Doxy, LH: Late harvest-High doxy), see also Fig 3B. **(C)** Viability staining showing surviving DIE cells containing single knockouts of potentials hits, identified in the CRISPR screen. Knockouts of individual genes were generated by transfection of sgRNA; 6 days later, cells were left untreated or treated with 3 different concentrations of doxycycline (100, 250 and 1000ng/ml) for and incubated for an additional 48–96 hours prior to visualizing surviving cells. Data are representative of at least three independent experiments. **(D)** Viability staining showing the surviving DIE-ieGFP-Cas9 cells (DIE cells expressing Cas9 constitutively and contain doxycycline-inducible eGFP) with single knockouts of mediator complex subunits. Knockouts of individual genes were generated by transfection of sgRNA; 6 days later cells were treated for doxycycline (250ng/ml) for 24h and incubated for an additional 48–96 hours prior to visualizing surviving cells. Data are representative of at least three independent experiments. **(E)** FACS data showing GFP-positive cells in surviving populations of DIE-ieGFP-Cas9 (expressing constitutive Cas9 and doxycycline-inducible GFP). cells containing single knockouts as indicated. Knockouts of individual genes were generated by transfection of sgRNA; 6 days later, cells were treated with doxycycline (250ng/ml) for 24h prior to FACS analysis. DIE-ieGFP-Cas9 cells comprised of 42% of eGFP-positive cells after DUX4 knockout. rtTA, MED25, MED24 and MED16 knockouts displayed a lower percentage of eGFP-expressing cells, comprising between 1.2–4% of eGFP-expressing cells. Data are representative of at least three independent experiments.

Since potential hits that mitigated DUX4 toxicity were likely obscured by the large number of false-positive hits that resulted from Cas9-mediated elimination of either the DUX4 or the rtTA3 transgenes, we filtered the screen results to remove all hits located on the q-arm of chromosome 5, or the p-arm of chromosome 19 (Fig 4A). After analyzing individual guides for their apparent effectiveness in the genome-wide screen (instead of the group average), a list of potential hits emerged (p-value ≤ 0.05, Log2(fold change) ≥ 1) for each of the 4 screens. Fig 4B shows the number of potential hits that met these criteria for each screen and how many of these hits are shared between them (see S4 Table for the full list of hits). We focused further on hits that emerged in at least 3 out of the 4 screens. Hits were validated by performing individual knockouts in the DIE-Cas9 cells. Of these hits, only the knockout of MED25 increased cell survival (Fig 4C). MED25 is a subunit of Mediator, a large complex that functions as a bridge between transcription factors and the transcriptional machinery. This includes RNA polymerase II, needed for the transcription of all protein coding genes in eukaryotes (reviewed by Soutourina [65]). The rescue seen in doxycycline-induced DIE cells after MED25 knockout diminished upon higher doxycycline exposure, suggesting that loss of MED25 provided a partial rescue. Other genes belonging to the same mediator complex, that initially did not meet our criteria, were re-evaluated by lowering the parameters (P ≤ 0.05, fold change of ≥ 1.5), identifying several other subunits. When individual knockouts of these genes were performed, two more subunits of the Mediator-complex, MED16 and MED24 showed partial rescue (Fig 4D). A potential role for the Mediator complex in DUX4 signaling is supported by a physical interaction between DUX4 and different subunits of the Mediator complex [38].

The hits in our screen appeared to interfere with DUX4 expression rather than with the DUX4-induced toxicity. Loss of function of components of our doxycycline-inducible system allows DIE cells to survive in the presence of doxycycline. Therefore, we developed a method to visualize the functionality of components of our doxycycline-inducible system. This method will exclude false-positive hits that interfere with DUX4 expression. We focused on rtTA3 transgene that binds to the tetracycline response element (TRE) and thus controls DUX4 expression (Fig 1A). An inducible eGFP under control of TRE was introduced in DIE-Cas9 cells (DIE-ieGFP-Cas9), resulting in rtTA3/TRE-dependent eGFP expression. This allowed to identify cells that survive by interfering with TRE-mediated expression (and thus expression of DUX4): cells containing a guide whose target can mitigate the apoptotic phenotype without interfering with the inducible system should not only survive but also have similar levels of eGFP as DIE-Cas9 cells in which DUX4 was knocked out (S7 Fig). To examine eGFP expression, cells with a single gene knockout were analyzed by flow cytometry. FACS analysis revealed that knockout of Mediator genes resulted in reduced induction of eGFP expression in surviving DIE cells (Fig 4E). This suggests that knockout of Mediator-complex components interferes with the ability of rtTA3 to induce TRE-mediated transgene transcription. Effect of MED25 knockout appeared therefore a result of impaired DUX4 activation, rather than true modulation of DUX4 cytotoxicity.

In a recent study by Shadle and colleagues, a siRNA screen was performed targeting the “druggable” genome to identify pathways of DUX4 toxicity. The study revealed the MYC-mediated apoptotic pathway and the viral dsRNA-mediated innate immune response pathway to be involved in DUX4-induced apoptosis [66]. When we examined our data for enrichment of sgRNA sequences that target the genes identified in the Shadle study, we did not observe significant enrichment in our CRIPSR screen data of these sequences (S7 and S8 Figs). Technical differences, such as the screening method, the complete or partial loss of function of genes, the scale of the screens (druggable genome vs whole genome) and the different cellular backgrounds, most likely all attributed to the little correlation seen between the two studies.

Another recently published genome-wide CRISPR/Cas9 study, where a similar methodology was used in a DUX4-inducible, immortalized myoblast line, identified the HIF1 oxidative stress pathway as a modulator of DUX4-induced apoptosis [67]. These data, as well as previous reports, clearly demonstrated the role of the HIF1 hypoxia pathway in DUX4-mediated cytotoxicity [67–69]. Members of the HIF1 pathways were not identified in our screen (S8 Fig), and neither did the other 14 significantly enriched hits they identified in their study, apart from MED25, which may be a false positive in both datasets. This indicates that changes in this pathway are likely not the only DUX4-induced cellular changes that push cells towards apoptosis. The fact that the HIF1 pathway was not identified in our screen could also indicate differences in sensitivity to oxidative stress between cellular systems, as different cell types experience and respond to oxidative stress differently.

## Discussion

The development use of CRISPR/Cas9 to carry out genome-wide forward genetic screens by knocking out gene expression has allowed analysis of virtually any aspect of mammalian biology (reviewed by Yu et al. [70]). These screens are more sensitive and efficient methods than knocking down gene expression by RNA interference due to incomplete knock down of gene expression.

Here, we aimed to obtain insight into the downstream targets of a transcription factor, DUX4, that are responsible for its cellular toxicity. We hypothesized that inhibition of key downstream DUX4 effectors would slow or abrogate the cytotoxic process, and set out to identify such genes by performing a genome-wide CRISPR/Cas9 screen. We carried out 4 parallel screens with different levels of DUX4 expression as well as different recovery times after DUX4 induction. The goal of the screen was to identify targets that can mitigate DUX4-induced toxicity. The screen’s technical execution went very well and displayed high sensitivity and specificity toward candidate editing events that indeed mitigated cytotoxicity in our transgene model. We know the screen was exhaustive because we picked up rare chromosomal rearrangements. However, none of the obtained hits had a direct effect on the DUX4 downstream transcriptional network but seemed to specifically affect the experimental system itself. We noticed that the chromosomal location of the hits overlapped with the insertion sites of either the DUX4 transgene or the rtTA3 inducer of the tetracycline-inducible system responsible for DUX4 transgene induction.

The main contributor for the effect seen when targeting Cas9 to genes that map to either chromosome 5 or 19 is likely a rare Cas9-induced chromosomal truncation or deletion, that deletes the transgenes when targeted to the chromosomal arm in which they have integrated. Although these events appear to be rare, nearly all guides that targeted genes located on the chromosomal arm to which rtTA3 had integrated (5q), were robustly enriched, underscoring the sensitivity of this screening method.

Most remaining hits (that were located on sites other than where the transgenes were inserted) did not appear to affect DIE cell survival upon individual validation, but members of the Mediator complex did show a positive effect on survival. However, these mediator subunit genes seemed to generally suppress rtTA3-mediated transcription, so their mitigating effect was not mediated by specifically altering DUX4 cytotoxicity. We therefore concluded, based on the conditions used in this study, that there is no individual target (other than DUX4 itself) that, upon knockout, can strongly inhibit DUX4-induced cytotoxicity. Efforts should therefore be redirected to the direct modulation of DUX4.

While sgRNAs in our library only target protein-coding genes, we believe we would have picked up any mitigating non-coding RNAs as well, had they provided a strong rescue from the DUX4 cytotoxic effects. In that case, one would have expected to see a similar hotspot of sgRNAs on and around the true target sites, as we observed for MED16, where a hotspot of sgRNAs was observed on the p-arm of chromosome 19, corresponding to the location of MED16. Another hotspot can be seen on the q arm of chromosome 19, corresponding to the location of MED25.

Recent attempts at targeting DUX4 directly include systems that do not lead to DNA damage, such as the use of antisense morpholino oligonucleotides to target and knock down the DUX4 transcript [71]. In another study, a catalytically inactive Cas9 fused to a *Krüppel*-associated box (dCas9-KRAB) was used to target the promotor of DUX4, inducing epigenetic repression of DUX4 [72]. Both studies show the ability to successfully diminish DUX4 expression in patient-derived cells. However, the effect of these types of interference may be transient, which make them less ideal for long-term treatment of FSHD. When these types of targeting strategies are combined with a gene therapy approach, as reported by Rashnonejad et al. [73].

While our screen did not identify target genes that can mitigate DUX4 cytotoxicity, it does illustrate some important aspects that need to be considered when performing phenotypic CRISPR/Cas9 screens. One aspect is the large chromosomal truncations that can be induced by Cas9, a phenomenon also recently reported by Cullot et al. [74]. While these are rare events in a cell population, our results demonstrate that in a sufficiently sensitive screening system, they are robustly identified and can crowd other potential positive hits. Continued selection for the transgenes that mediate expression the screened phenotype (in our case of DUX4) will help in this aspect by removing cells that have lost their resistance marker (linked to the transgenes that mediate expression) is therefore essential. Another aspect that needs consideration are the endogenous genes that have a general effect on transcription and translation, in this case effecting the inducible system, like subunits of the mediator complex identified in this study. Potential hits will always need to be validated individually in such a way that can exclude this possibility, like shown here, or by Shadle et al., where some of the same genes were identified affecting their inducible Tet-On system [66].

Our findings demonstrate the importance of including controls into the screen setup to ensure that the different components to carry out CRISPR/ Cas9 knockout screens are intact, and thus exclude potential false positives. We have provided a control to exclude false-positives that visualizes whether rtTA3 can still induce TRE-mediated expression (to induce DUX4 expression) by verifying expression of eGFP that is also under the control of TRE. By using this control, we were able to demonstrate that the attenuating effect of genes of the MED25 on survival was a result of a decrease in rtTA3-mediated expression (as measured by a decrease in eGFP levels).

This study started out with the aim of trying to contribute to the understanding of the underlying molecular mechanisms of FSHD, by performing a genome-wide CRISPR-Cas9 phenotypic screen. However, with no significant hits that can explain their contribution to the apoptotic phenotype, this story also tells a cautionary tale for knockout screens with CRISPR-Cas9. Our data, and suggestions to circumvent potential false-positives, will benefit future groups planning to execute similar screens.

## Methods

### Cloning and generating the DIE cell line

To generate the inducible DsRed/DUX4 system, the third generation lenti-viral plasmid pRRLsincPPT-wpre [75] was used as the backbone. The linearized viral backbone was created by restriction digestion using the following enzymes: HpaI and SalI (NEB). All inserts were generated with PCR amplification using phusion DNA polymerase (Fischer Scientific). Insert were created with 15bp adapter sequences, matching the backbone or neighboring fragments, for in-fusion cloning (Clontech). The first fragment consisted of cPPT/CTS-TRE-mCMV sequences, and the second fragment contained the LoxP-DsRed-LoxP (LSL) sequence. After inserting these two fragments into the pRRLsincPPT-wpre backbone, this newly cloned construct was transformed into chemically competent Stbl3 Escherichia coli (E.coli). The plasmid was isolated and purified from the Stbl3 cells using the HiPure plasmid kits from Invitrogen (Fischer scientific). This TRE-LSL plasmid was then opened up using restriction enzymes XbaI and EcoRI (NEB), after which the remaining three inserts: DUX4(exon1-3), mPGK and PuroR-WPRE, were cloned downstream from the LoxP-DsRed-LoxP in similar fashion.

The DIE cell line was obtained by transducing diploid KBM7 cells with lentiviral particles containing the inducible DsRed/DUX4 cassette mentioned above. 2 days after lentiviral transduction, transfected cells were selected with puromycin. After establishing a stable line by puromycin selection, lentiviral particles containing CMV-rtTA3-BlastR were added to the DsRed/DUX4-containing KBM7 cells. Positively transfected cells were subsequently selected with blasticidin, and FACS-sorted for DsRed expression upon exposure to doxycycline. The pLenti CMV rtTA3 Blast (w756-1) was a gift from Eric Campeau (Addgene plasmid #26429).

### Cell culture

KBM7 cells were cultured in IMDM media (Fischer Scientific) supplemented with 10% FBS. The DIE cells were cultured in IMDM media supplemented with 10% Tet system approved FBS (Clontech), 5μg/ml Puromycin and 6μg/ml Blasticidin, 100μM 2-mercaptoethanol.

### Cloning p2T-Cas9, p2T-ieGFP and sgRNA constructs, and generating DIE-Cas9 and DIE-Cas9-ieGFP cell lines

The p2T-CAG-spCas9-NeoR mammalian expression plasmid was created by replacing the Blasticidin resistance gene (BlastR) in the p2T-CAG-spCas9-BlastR (Addgene: 107190) [76] with a Neomycin resistance gene (NeoR). The p2T-CAG-spCas9-BlastR plasmid is contained in a p2Tol2 backbone [77]. The BlastR gene was removed using restriction digestion, using MfeI and AflII (NEB). Cloning the NeoR DNA fragment into the p2T-CAG-spCas9 backbone was done in similar fashion as described above. The p2T-CAG-SpCas9-BlastR was a gift from Richard Sherwood. The p2T-TetO-eGFP-HygroR plasmid was generated in a similar way as the p2T-CAG-spCas9-NeoR. In short, all sequences between transposable elements of a p2T plasmid were removed by restriction digestion using AleI and EcoRI (NEB). The TetO-eGFP-HygroR cassette was created by amplifying each subunit individually from already excising constructs, and thereafter cloned into the empty p2T backbone, using in-fusion cloning.

Both p2T-CAG-spCas9-NeoR and p2T-TetO-eGFP-HygroR were introduced in the DIE cell line by using Transposase. The p2T-CAG-spCas9-NeoR was introduced into DIE cells together with a plasmid encoding for transposase, using Polyethylenimine (PEI) transfection reagent (4ug PEI per 1ug DNA). The DIE cells were exposed to the transfection mixture for 14-16h, after which the transfection media was replaced with growth media. Geneticin g418 selection was started two days post transfection, generating the DIE-Cas9 line. The DIE-Cas9-ieGFP cell line was created by adding Transposase and p2T-TetO-eGFP-HygroR the DIE-Cas9 line, described as above.

spCas9-sgRNA constructs were cloned using a plasmid containing a U6 promotor, 2 BsmBI sites directly adjacent to the tracrRNA sequence, and a Hygromycin resistance gene (made in-house). This U6-2xBsmBI-Tracr-HygroR plasmid was digested with the BsmBI restriction enzymes (NEB), after which the CRISPR inserts were ligated in using T4 DNA ligase (NEB). CRISPR inserts were generated by annealing two complementary oligos containing a 4bp adapter serving as the BsmBI sticky end.

All plasmids mentioned in this study were transformed in chemically competent Stbl3 Escherichia coli (E. coli), and prepped using a HiPure plasmid Midi or Maxi kit (Invitrogen).

### RNA extraction and RT-qPCR

Cultured cells were rinsed with DPBS just prior to the additional of TRIzol reagent (Thermo Scientific), and stored at -80°C until further processing. Total RNA samples were subsequently extracted by addition of chloroform, and isopropanol precipitation, and finally treated with RNase free DNase I (Promega). Reverse transcription was performed using the Superscript III kit (Invitrogen) and random primers (Promega), generating cDNA. Quantitative PCR was then initiated using IQ SYBR Green Supermix (Bio-Rad 1708880), 50 ng of cDNA, and the following gene-specific primers:

- DUX4: 5’-CCCAGGTACCAGCAGACC-3’,

5’-TCCAGGAGATGTAACTCTAATCCA-3’ [78];

- ZSCAN4: 5’-GTGGCCACTGCAATGACAA-3’,

5’-AGCTTCCTGTCCCTGCATGT-3’ [78];

- ZNF217: 5’-AAGCCCTATGGTGGCTCC-3’,

5’-TTGATATGACACAGGCCTTTTTC-3 [78]’;

- PRAMEF1: 5’-CTCCAAGGACGGTTAGTTGC-3’,

5’-AGTTCTCCAAGGGGTTCTGG-3 [78]’;

- LEUTX: 5’- GGCCACGCACAAGATTTCTC-3’,

5’- TCTTGAACCAGATCTTTACTACGGA-3’;

- GAPDH: 5’-TCCAAAATCAAGTGGGGCGA-3’,

5’-AAATGAGCCCCAGCCTTCTC-3’;

- 18S: 5’-GTAACCCGTTGAACCCCATT-3,

5’-CCATCCAATCGGTAGTAGCG-3

- HPRT: 5’- CCTGGCGTCGTGATTAGTGA-3’,

5’- CGAGCAAGACGTTCAGTCCT-3’ [79].

A two-step qPCR program was initiated with a denaturing temperature of 95°C, and an annealing and elongation temperature of 95°C. A melt curve analysis was performed at the end of the 40 amplification cycles, from 55 to 95°C with increasing increments of 0.5°C. All samples were generated in triplicate, and with experiment being repeated 3x (n = 3). No template (blanc) controls of each primer set were also taken along in triplicate, with each experiment. Three loading controls (18S, GAPDH, and HPRT) were tested for their effect on expression between treated and untreated samples. The loading control that showed minimal difference in Ct value between all samples (S2 Fig) was chosen for data normalization. Data was normalized to HPRT expression by using the following primer pair: 5’- CCTGGCGTCGTGATTAGTGA-3’, 5’- CGAGCAAGACGTTCAGTCCT-3’ [79]. All samples with a Ct value higher than 35 were not included in the data analysis. All samples demonstrating multiple peaks in their melting curve were also removed before analysis. The reference genes HPRT, GAPDH and 18S displayed no change in Ct values upon doxycycline treatment (S2 Fig). Data were normalized to HPRT expression and are displayed as fold change (2^-ddCt) between untreated and doxycycline-treated samples. Data were analyzed by ANOVA using Prism software.

### Protein extraction and western blot

DIE cells were harvested by trypsinization and lysed with RIPA buffer. Total protein concentrations were determined using a Pierce BCA protein assay kit (Fischer Scientific). 20ug protein was denatured using 4x Laemmli sample buffer (Bio-Rad) with 10% BME (Sigma), and boiled for 5 minutes. Samples were run on a 15% SDS-polyacrylamide gel and transferred to a PVDF membrane (Merck). Membranes were blocked for an hour using 5% BSA in TBST, and were subsequently incubated overnight with anti-DUX4 antibody [E5-5] (Abcam, ab124699) in blocking solution (5% BSA in TBST), at 4°C. Membranes were than incubated for an hour with secondary goat anti-rabbit-HRP antibody (Santa Cruz, sc-2004), and primary rabbit mAb β-Actin HRP conjugated antibody (Cell signaling, 5125s) in blocking buffer. Chemiluminescent signal was detected using GE ImageQuant LAS 4000 imager, upon admission of Pierce ECL Plus Western Blotting substrate (Fischer Scientific).

### RNAseq sample preparation and sequencing

Cultured cells were rinsed with DPBS just prior to the additional of TRIzol reagent (Thermo Scientific). Total RNA samples were subsequently extracted by addition chloroform, and isopropanol precipitation. The library prep was performed using CEL-seq1 primers [80] and the Life technologies Ambion kit (AM1751) [81], and were processed using CEL-seq2 protocol [82]. Samples were sequenced using Illumina Nextseq 500, 2x75 kit, high output. Four technical replicates per samples were sent for sequencing, and were sequenced to an average of 600,000 reads per replicate (combined read count of 2.4 million reads per sample). Differential expression analysis was done using the DESeq2 package [83].

### Doxycycline titration curve

200,000 cells were seeded into wells of a 24-wells plate and incubated overnight at 5% CO_2,_ at 37°C. When cells reached a density of 90–100% confluency, different concentrations of doxycycline were added to the vertical lanes (100ng/ml, 250ng/ml, 500ng/ml, 750ng/ml, 1000ng/ml), with the horizontal lanes experiencing different exposure times (48h, 36h, 24h, 12h). After a recovery period of 96h (after doxy exposure was ended), cells were washed with DPBS, and fixed with Methanol for 10 minutes. Giemsa stain, modified solution (Sigma) was subsequently added for 45 minutes, after which it was removed, and the wells were washed with demineralized water.

### Genome-wide CRISPR screen

The screen on the DIE line was performed as previously described by Doench et al. [43] and Sanson et al. [84]. Due to a shared selection marker between the DIE line and the all-in-one Brunello lentiviral library, transfected cells could not be selected for, thus the total number of cells was raised to 1500 cells per guide, when considering an average transfection efficiency of 30–50% in all cell lines tested by Doench et al. [43]. To minimize the probability of multiple sgRNA plasmids entering one cell, we determined the transfection efficiency and calculated the MOI. With 1500 cells per guide (total of 77,441 guides), each of the three replicates contained 120*10E6 cells. These cells were spin transfected for 2h at 1000g with 82*10E6 Brunello virus particles (LentiCRISPRv2, Addgene 73179-LV, all-in-one system in which every plasmid contains SpCas9, and a guide RNA) reaching a MOI of 0.65, and a transfection efficiency of around 60% upon testing the viral library on the KBM7 parental line. After transfection the 120*10E6 transfected cells (contained in 40 wells of 12-well tissue culturing plates) were trypsinized and passaged to 60 145mm TC plates. Mutagenized cells were maintained for 6 days, before inducing a set of 24 plates with either a low or high doxycycline concentration (low: 250ng/ml, high: 1000ng/ml). The remaining 12 plates were harvested for cryofreezing (7 plates) and for determining library coverage (5 plates). After a 24h doxycycline induction period, 12 plates were given a 24h recovery period (early harvest) of both the low and high doxycycline-exposed sets. The remaining 24 plates received an additional 48h of recovery time (late harvest), before harvesting the surviving cells for sequencing (S1 Fig). Cell Pellets were stored at -80°C until further processing. The Human Brunello CRISPR knockout pooled lentiviral prep library was a gift from David Root and John Doench (Broad Institute, MA, U.S.A.).

### Library prep, sequencing and analysis

Genomic DNA (gDNA) was isolated using NucleoSpin Blood Mini (less than 5 million cells), Midi (L) (5–20 million cells) and Maxi (XL) (more than 20 million cells) kits, depending on the size of cell pellet. Libraries were prepared and sequenced on a HiSeq2000 (Illumina) as described by Doench et al. Analysis was conducted using “STARS”, gene-ranking method to generate FDR values developed by Doench et al. that was used to generate p-values and FDR rates [43]. Chromosomal ideogram were generated by using the phenoGram webtool from the Ritchie lab from the university of Pennsylvania [85].

### Individual knock outs cell survival Giemsa staining

DIE-Cas9 and DIE-Cas9-ieGFP were seeded in 24-well plates. The next day, when the cells had reached 70–90% confluency, cells were transfected with 500ng guide plasmid per well using 4ug PEI per 1ug DNA. During the overnight transfection no selection markers were present in the media, however growth media was supplemented with 100U/ml pen-strep. Cells were passaged with or without selection markers during a period of 6–7 days, after which doxycycline was added (100, 250 or 1000 ng/ml) for a 24h period. Wells were washed with DPBS to remove dead cells and debris. Remaining cells were given the opportunity to grow out, or to die (if they had already entered the apoptotic pathway) for an additional 48–96 hours. The wells were stained using Giemsa modified solution, as described previously.

### Flowcytometry sorting (FACS) and analysis

DIE-Cas9-ieGFP cells were induced with 250ng/ml doxycycline 24h prior to FACS analysis. After the 24h doxycycline exposure, cells were trypsinized using 0.25% Trypsin-EDTA, resuspended in iMDM media supplemented with Tet approved FBS and DAPI nuclear staining, and strained using a Cell-strainer capped tubes (Falcon). Cells were analyzed using the Beckman coulter Cytoflex S flow cytometer.

### Imaging DIE cells

Untreated and doxycycline-treated DIE cells were stained with AnnexinV-Alexa Fluor 488 and PI (Thermo Scientific), by adding the staining solutions directly to growth medium at a 1:50 and 1:100 ratio respectively. Cells were incubated overnight in growth medium containing staining solution(s) and with or without doxycycline treatment. Live imaging during treatment of these cells was carried out using a Confocal microscopy (Zeiss LSM 700). Still images were taken after the overnight incubation with an EVOS Digital Color Fluorescence Microscope (Invitrogen).

## Supporting information

S1 Fig Execution of the CRIPSR/Cas9 genome-wide screens. A schematic representation of the execution of the CRISPR/Cas9 knockout screens. PB: Polybrene, TC: Tissue culture, LE: Low doxycycline/Early harvest, HE: High doxycycline/Early harvest, LL: Low doxycycline/Late harvest, HL: High doxycycline/Late harvest (see also Fig 3B), Library rep: Library representation.
(PDF)

S2 Fig Analysis of apoptosis in DIE cells and expression of reference genes following doxycycline treatment. **(A)** Uninduced (top panel) and doxycycline-induced (bottom panel) DIE cells, stained with Propidium Iodide (PI) (middle panel) and AnnexinV-Alexa Fluor 488 (right panel), with a phase contrast image in the left panel. DIE cells in the bottom panel are stained positive for AnnexinV, with no increasing PI signal compared to uninduced DIE cells (top panel). **(B)** Analysis of the fraction of viable cells with and without doxycycline treatment (1000ng/ml). **(C)** Expression of HPRT, GAPDH and 18S in KBM7 cells or in DIE cells with and without doxycycline treatment, analyzed by qRT-PCR. Data were analyzed by ANOVA analysis. **(D)** HPRT Ct values of KBM7 cells, DIE cells and DIE KO cells that were untreated or treated with doxycycline (1000ng/ml). These values were used to normalize induction of gene expression in Fig 1F. Doxycycline did not significantly affect HPRT expression in the different cell lines that were tested. Data were analyzed by ANOVA analysis.
(PDF)

S3 Fig Analysis of enriched sgRNAs from screen data processed with a one-sided analysis. Volcano plots illustrating enrichment of sgRNAs in the surviving population of DIE cells of all 4 screens (see Fig 3B). Due to the one-sided analysis, depletion data should not be taken into consideration. For a two-sided analysis see S6 Fig. The Log2(fold change) (log2FC) is plotted on the X-axis and the -Log10(p-value), (-log10PV) is plotted on the Y-axis. Data shown here show the average log2FC and -log10PV of each guide set (set: 4 guides per gene). Blue points represent guide sets that are significantly enriched in this data set (Log2FC ≥ 1, -log10PV ≥ 2), purple points represent the false-positive hits that on chromosome 5q and chromosome 19p, green point are the positive controls (DUX4, MAST1, MGAT4B), red points represent the Non-Target control guides.
(PDF)

S4 Fig PhenoGrams showing enriched hits in the human genome. Chromosomal ideogram indicating the location of enriched hits in the human genome, for each of the 4 screens. PhenoGram is a software created by the Ritchie lab from the university of Pennsylvania [85].
(PDF)

S5 Fig Data plot displaying enriched hits on chromosome 5q and chromosome 19. The average-Log(p-value) is plotted on the Y-axis, and the X-axis is displaying the position on the chromosome. The vertical abline indicates the position of the centromere. All points above the horizontal abline (in blue) indicating significantly enriched hits that fall below the 5% False Discovery Rate (FDR) threshold. The location of the transgene is annotated with a blue arrow on the X-axis.
(PDF)

S6 Fig Individual knock-outs in DIE-ieGFP-Cas9 cells demonstrating eGFP activation in cells with a functional TetO-inducible system. Phase contrast (top panel) and fluorescent images (bottom panel) of DIE-ieGFP cells containing a DUX4 KO (left panel), and rtTA3 KO (right panel) induced with 250 ng/ml doxycycline.
(PDF)

S7 Fig Validation of genes involved in the MYC-mediated apoptotic pathway and the viral dsRNA-mediated innate immune response pathway. **(A)** Data plots showing the significance and enrichment of sgRNAs targeting DUX4, MED25, RPS25 and CISD, in all 4 screens. The Log2(fold-change) (L2FC) of each individual guide is plotted on the left y-axis indicated in blue, and the–Log10(P-value) is plotted on the right y-axis, in red. When guides fall above the blue and red intermitted ablines, they are considered significant (Log2(fold change) > 1, -Log10(P-value) > 1.3). The sgRNAs that are significantly enriched in all 4 screens are underlined. All 4 sgRNAs targeting DUX4 are significantly enriched. 3 out of 4 sgRNAs targeting MED25 are significantly enriched (guides 1, 2 and 3). sgRNAs 1 and 4 targeting PRS25 are significantly enriched, and CISD has one sgRNA that is significantly enriched in all 4 screens. **(B)** Data plots showing the enrichment of sgRNAs targeting FOSB, RNASEL, MYC, FXN and EAF1. None of the 4 guides show significant enrichment in any of the 4 screens. **(C)** Viability staining showing surviving DIE- Cas9 cells (DIE cells constitutively expressing Cas9) containing single knockouts of genes involved in the MYC-mediated apoptotic pathway and the dsRNA-mediated immune response pathway (Top panel). Controls can be found in the bottom panel and are as followed, positive controls: DUX4, rtTA3, MED24, MED16 and MED25; Negative non-target control: NT.
(PDF)

S8 Fig Validation of genes involved in the HIF1 hypoxia pathway. **(A)** Data plots showing the enrichment of sgRNA targeting HIF1A. HIF1B/ARNT, CDKN1A. The LFC value of each individual guide is plotted on the left y-axis, indicated in blue, and the–Log10 P-value is plotted on the right y-axis, in red. Guides located above the blue and red intermitted ablines are considered significant (blue: LFC > 1, red: -Log10 P-value > 1.3). **(B)** Viability staining of untreated DIE-Cas9 cells (top panel) and treated with 1000ng/ml doxycycline (lower panel), transfected with DUX4, rtTA3, HIF1A and non-targeting (NT) sgRNA-coding plasmids.
(PDF)

S1 Table Differentially expressed genes after 4.5h of doxycycline induction. (XLSX)

S2 Table TF perturbations followed by expression. (XLSX)

S3 Table Shared differentially upregulated genes between 8.5h induced DIE cells and other datasets. (XLSX)

S4 Table Enriched sgRNAs and their corresponding genes. (XLSX)

S1 Video Effect of doxycycline on KBM7 cells. KBM7 cells were treated with doxycycline (100ng/ml) and imaged for 12 hours to visualize AnnexinV staining.
(MP4)

S2 Video DIE cells without doxycycline. DIE cells were left untreated and imaged for 12 hours to visualize AnnexinV staining.
(MP4)

S3 Video Effect of doxycycline on DIE cells. DIE cells were treated with doxycycline (100ng/ml) and imaged for 12 hours to visualize AnnexinV staining.
(MP4)

S1 File (DOCX)
