# Supplementary figures and images for: Considerations and practical implications of performing a phenotypic CRISPR/Cas survival screen
Source: PLoS One. 2022 Feb 17;17(2):e0263262. doi: 10.1371/journal.pone.0263262 (PMC8853573; doi:10.1371/journal.pone.0263262)

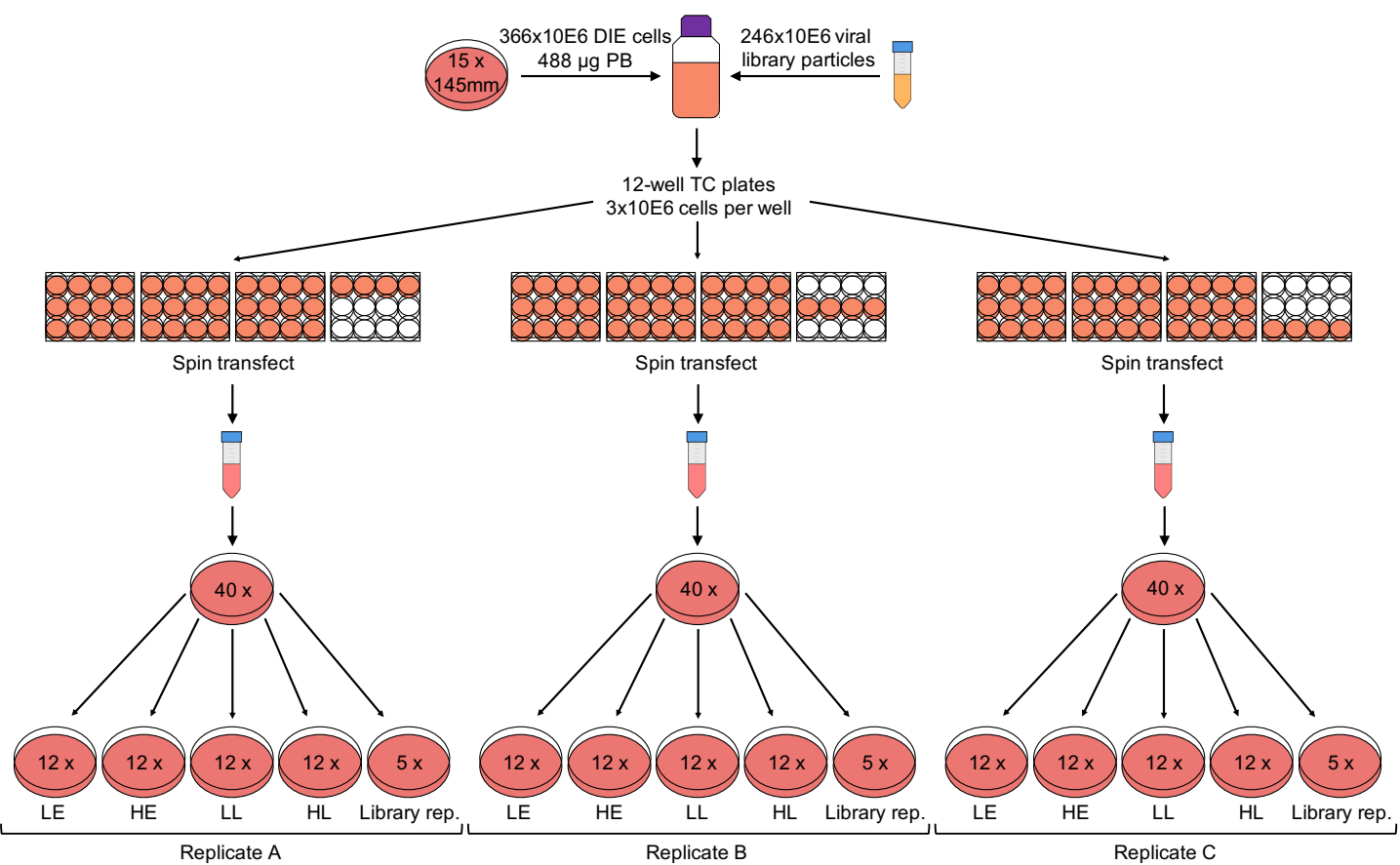

Supplement: S1 Fig — A schematic representation of the execution of the CRISPR/Cas9 knockout screens. PB: Polybrene, TC: Tissue culture, LE: Low doxycycline/Early harvest, HE: High doxycycline/Early harvest, LL: Low doxycycline/Late harvest, HL: High doxycycline/Late harvest (see also Fig 3B), Library rep: Library representation. (PDF) [file pone.0263262.s001.pdf]

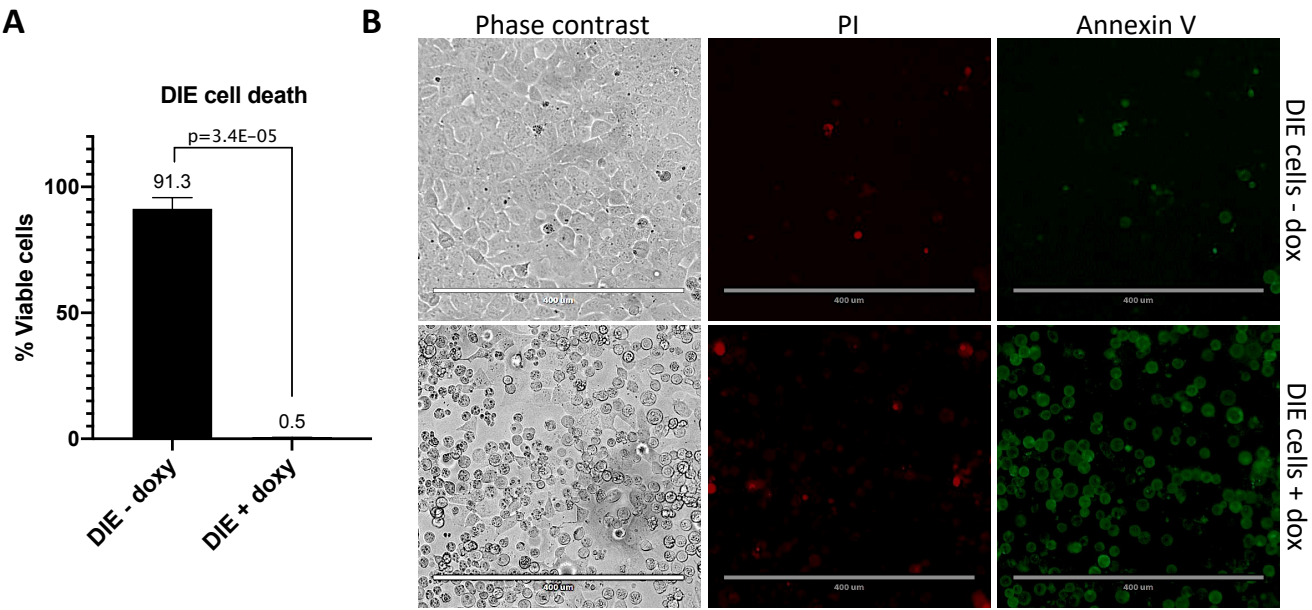

Supplement: S2 Fig — (A) Uninduced (top panel) and doxycycline-induced (bottom panel) DIE cells, stained with Propidium Iodide (PI) (middle panel) and AnnexinV-Alexa Fluor 488 (right panel), with a phase contrast image in the left panel. DIE cells in the bottom panel are stained positive for AnnexinV, with no increasing PI signal compared to uninduced DIE cells (top panel). (B) Analysis of the fraction of viable cells with and without doxycycline treatment (1000ng/ml). (C) Expression of HPRT, GAPDH and 18S in KBM7 cells or in DIE cells with and without doxycycline treatment, analyzed by qRT-PCR. Data were analyzed by ANOVA analysis. (D) HPRT Ct values of KBM7 cells, DIE cells and DIE KO cells that were untreated or treated with doxycycline (1000ng/ml). These values were used to normalize induction of gene expression in Fig 1F. Doxycycline did not significantly affect HPRT expression in the different cell lines that were tested. Data were analyzed by ANOVA analysis. (PDF) [file pone.0263262.s002.pdf]

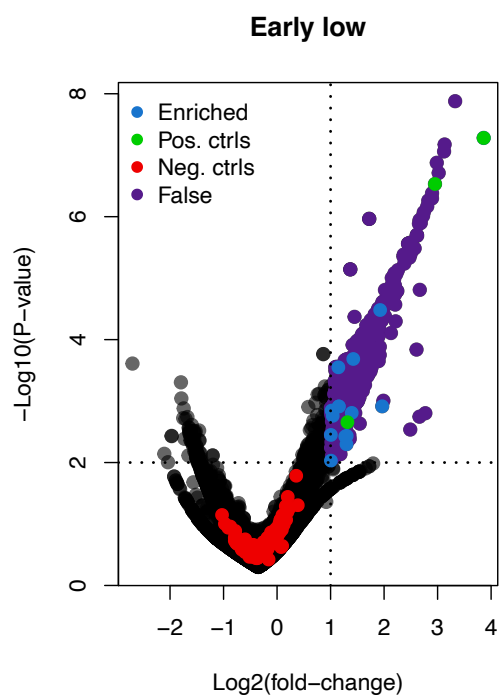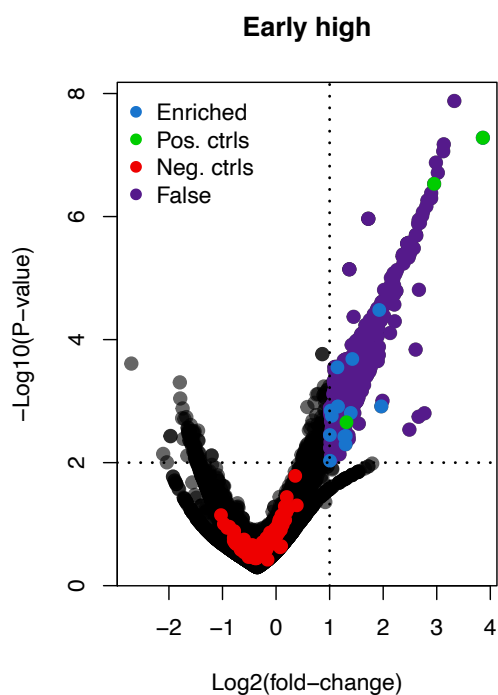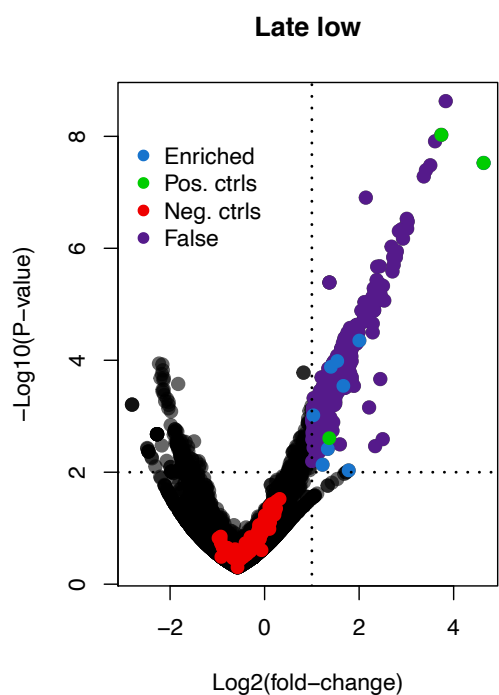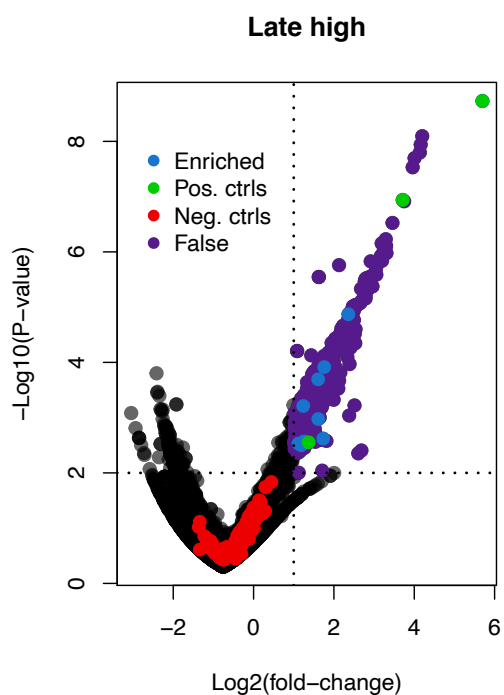

Supplement: S3 Fig — Volcano plots illustrating enrichment of sgRNAs in the surviving population of DIE cells of all 4 screens (see Fig 3B). Due to the one-sided analysis, depletion data should not be taken into consideration. For a two-sided analysis see S6 Fig. The Log2(fold change) (log2FC) is plotted on the X-axis and the -Log10(p-value), (-log10PV) is plotted on the Y-axis. Data shown here show the average log2FC and -log10PV of each guide set (set: 4 guides per gene). Blue points represent guide sets that are significantly enriched in this data set (Log2FC ≥ 1, -log10PV ≥ 2), purple points represent the false-positive hits that on chromosome 5q and chromosome 19p, green point are the positive controls (DUX4, MAST1, MGAT4B), red points represent the Non-Target control guides. (PDF) [file pone.0263262.s003.pdf]

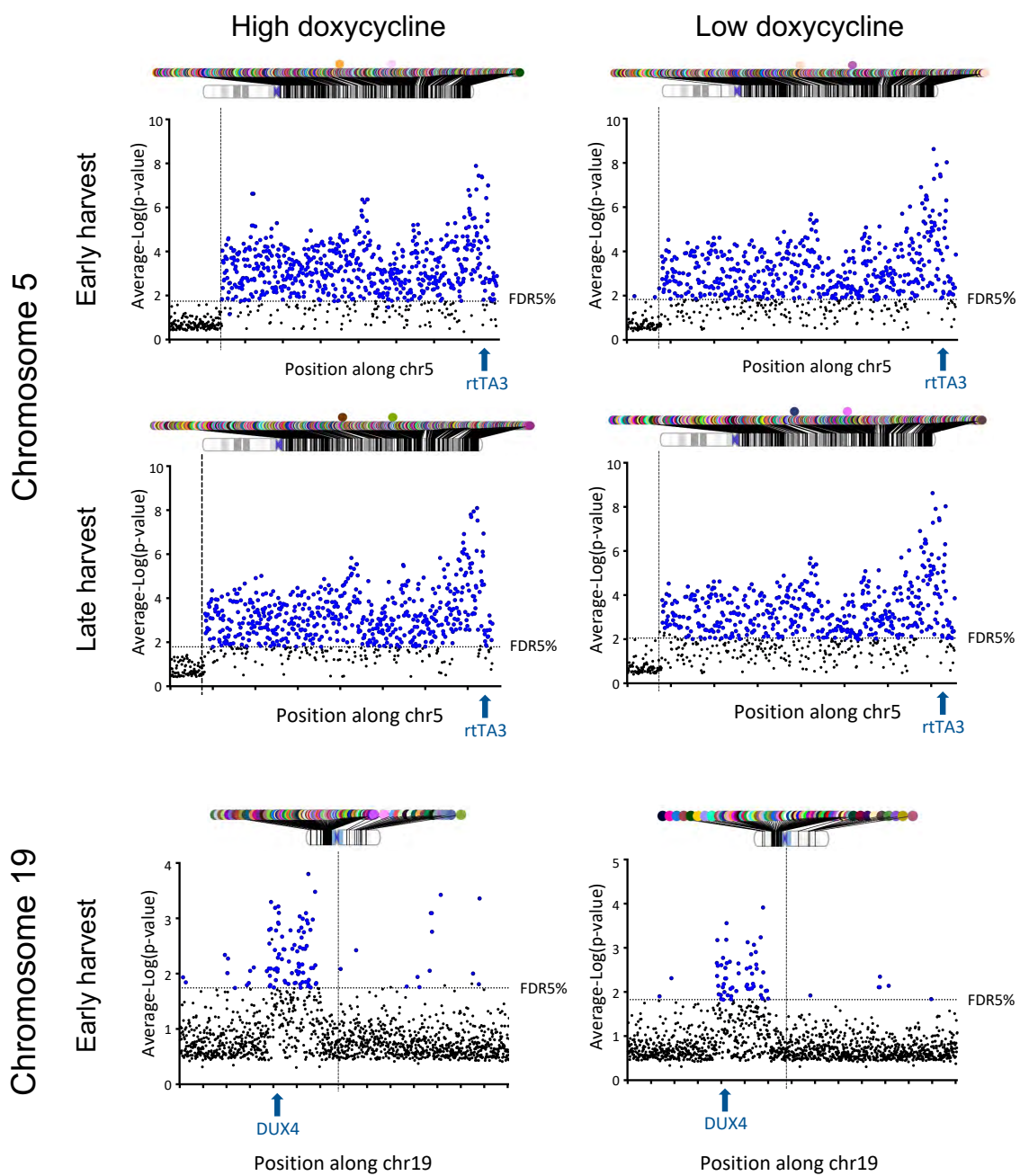

Supplement: S5 Fig — The average-Log(p-value) is plotted on the Y-axis, and the X-axis is displaying the position on the chromosome. The vertical abline indicates the position of the centromere. All points above the horizontal abline (in blue) indicating significantly enriched hits that fall below the 5% False Discovery Rate (FDR) threshold. The location of the transgene is annotated with a blue arrow on the X-axis. (PDF) [file pone.0263262.s005.pdf]

DUX4 KO

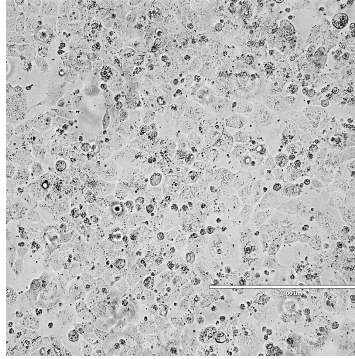

rtTA3 KO

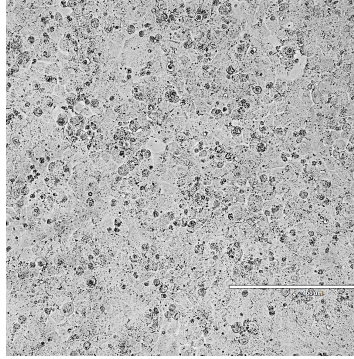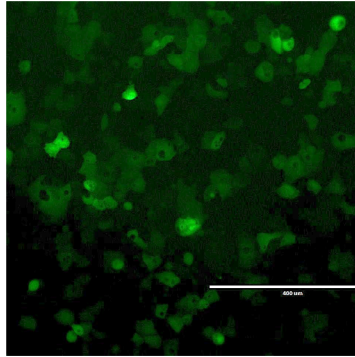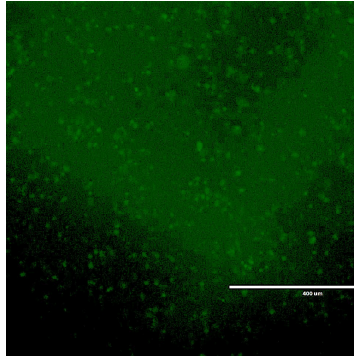

Supplement: S6 Fig — Phase contrast (top panel) and fluorescent images (bottom panel) of DIE-ieGFP cells containing a DUX4 KO (left panel), and rtTA3 KO (right panel) induced with 250 ng/ml doxycycline. (PDF) [file pone.0263262.s006.pdf]

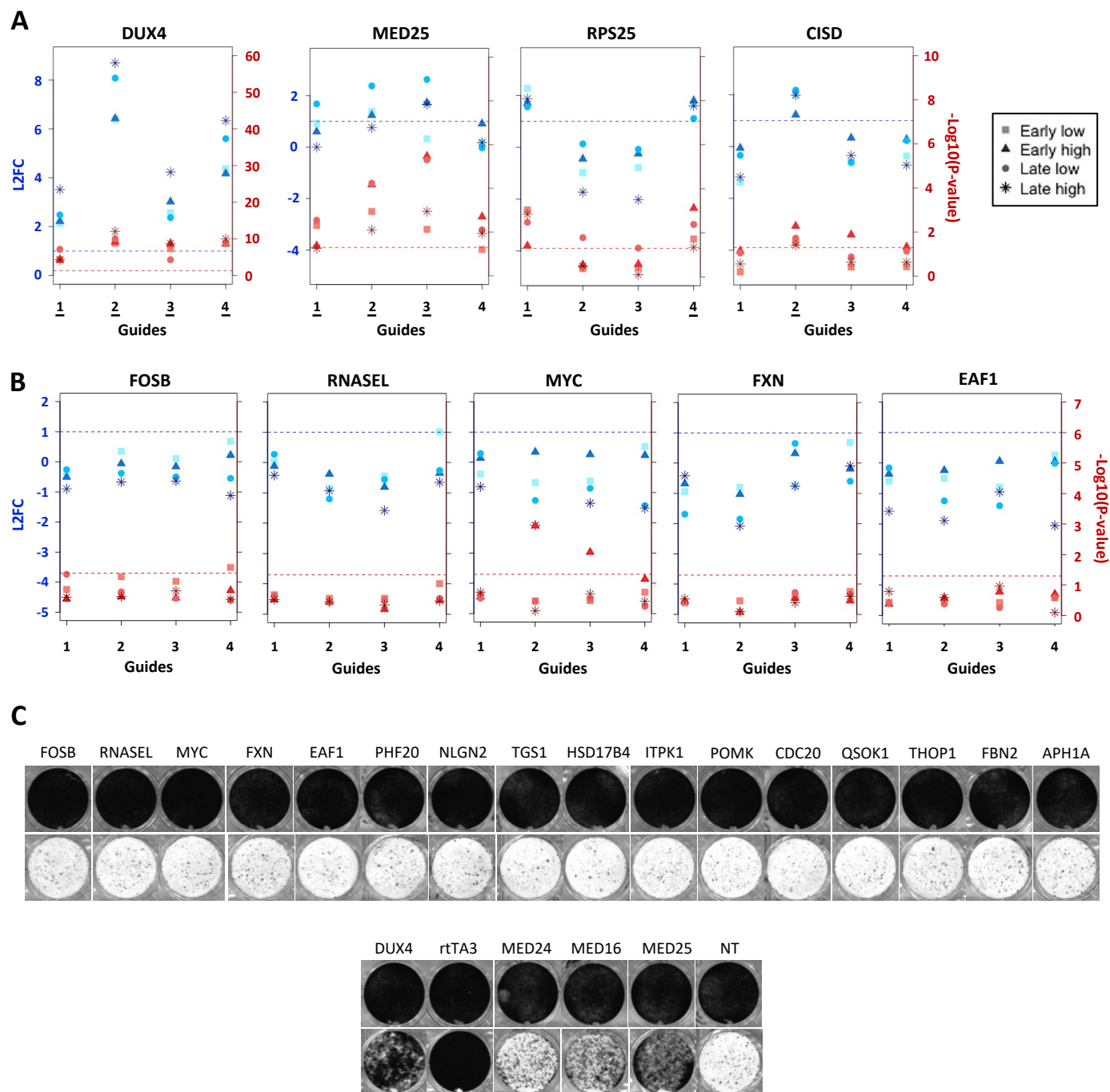

Supplement: S7 Fig — (A) Data plots showing the significance and enrichment of sgRNAs targeting DUX4, MED25, RPS25 and CISD, in all 4 screens. The Log2(fold-change) (L2FC) of each individual guide is plotted on the left y-axis indicated in blue, and the–Log10(P-value) is plotted on the right y-axis, in red. When guides fall above the blue and red intermitted ablines, they are considered significant (Log2(fold change) > 1, -Log10(P-value) > 1.3). The sgRNAs that are significantly enriched in all 4 screens are underlined. All 4 sgRNAs targeting DUX4 are significantly enriched. 3 out of 4 sgRNAs targeting MED25 are significantly enriched (guides 1, 2 and 3). sgRNAs 1 and 4 targeting PRS25 are significantly enriched, and CISD has one sgRNA that is significantly enriched in all 4 screens. (B) Data plots showing the enrichment of sgRNAs targeting FOSB, RNASEL, MYC, FXN and EAF1. None of the 4 guides show significant enrichment in any of the 4 screens. (C) Viability staining showing surviving DIE- Cas9 cells (DIE cells constitutively expressing Cas9) containing single knockouts of genes involved in the MYC-mediated apoptotic pathway and the dsRNA-mediated immune response pathway (Top panel). Controls can be found in the bottom panel and are as followed, positive controls: DUX4, rtTA3, MED24, MED16 and MED25; Negative non-target control: NT. (PDF) [file pone.0263262.s007.pdf]

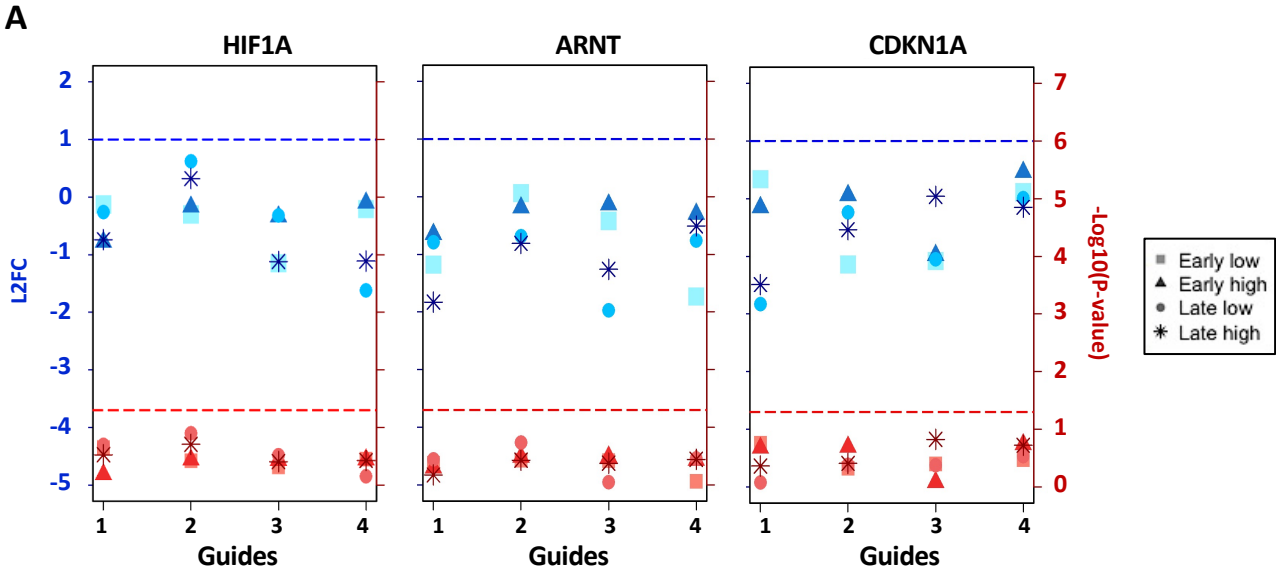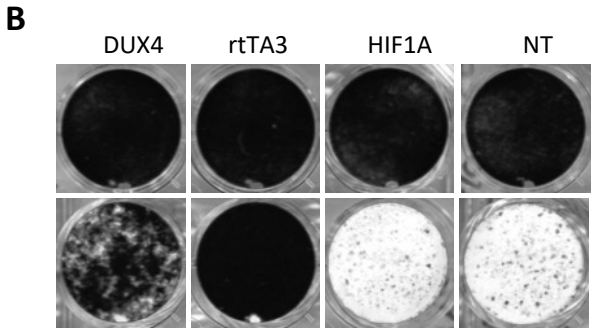

Supplement: S8 Fig — (A) Data plots showing the enrichment of sgRNA targeting HIF1A. HIF1B/ARNT, CDKN1A. The LFC value of each individual guide is plotted on the left y-axis, indicated in blue, and the–Log10 P-value is plotted on the right y-axis, in red. Guides located above the blue and red intermitted ablines are considered significant (blue: LFC > 1, red: -Log10 P-value > 1.3). (B) Viability staining of untreated DIE-Cas9 cells (top panel) and treated with 1000ng/ml doxycycline (lower panel), transfected with DUX4, rtTA3, HIF1A and non-targeting (NT) sgRNA-coding plasmids. (PDF) [file pone.0263262.s008.pdf]
