# Supplementary material for: Considerations and practical implications of performing a phenotypic CRISPR/Cas survival screen
Source: PLoS One. 2022 Feb 17;17(2):e0263262. doi: 10.1371/journal.pone.0263262 (PMC8853573; doi:10.1371/journal.pone.0263262)
Supplement: S4 Fig — Chromosomal ideogram indicating the location of enriched hits in the human genome, for each of the 4 screens. PhenoGram is a software created by the Ritchie lab from the university of Pennsylvania [85]. (PDF) [file pone.0263262.s004.pdf]

Early low

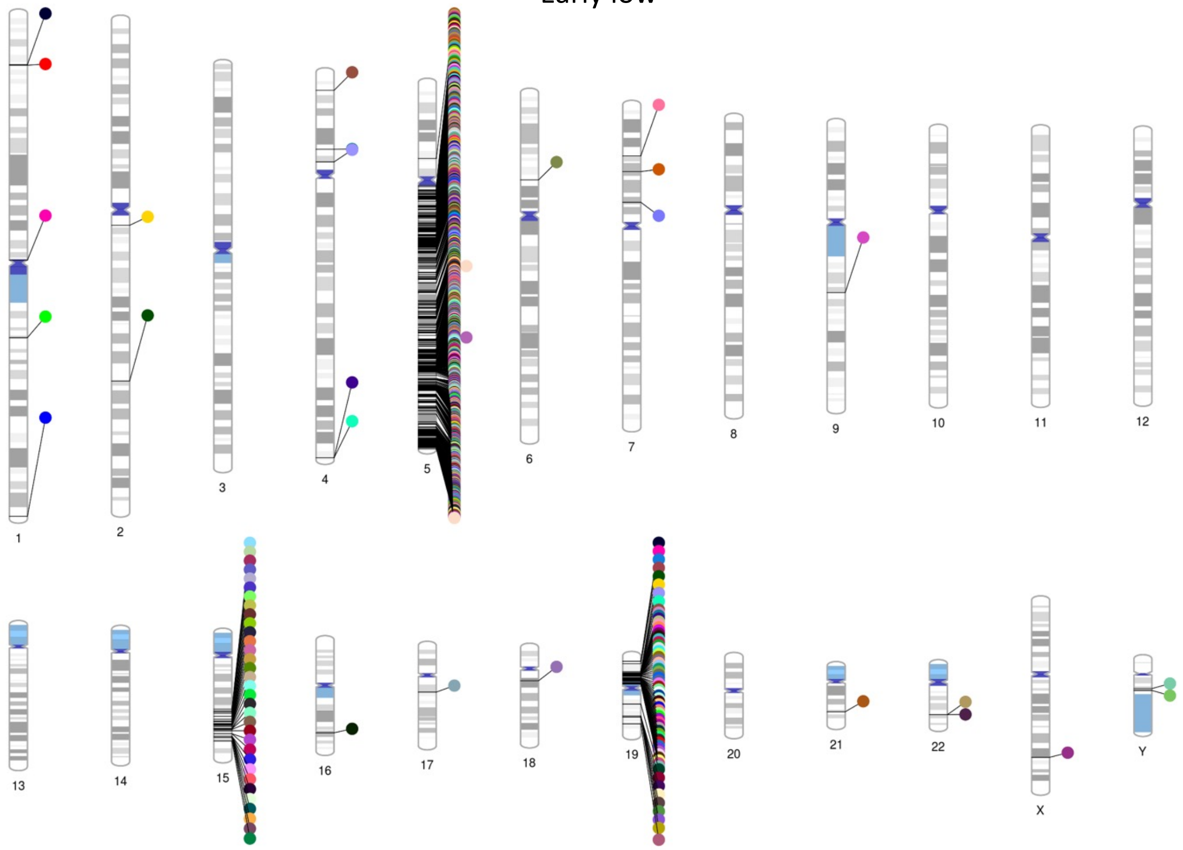

Early high

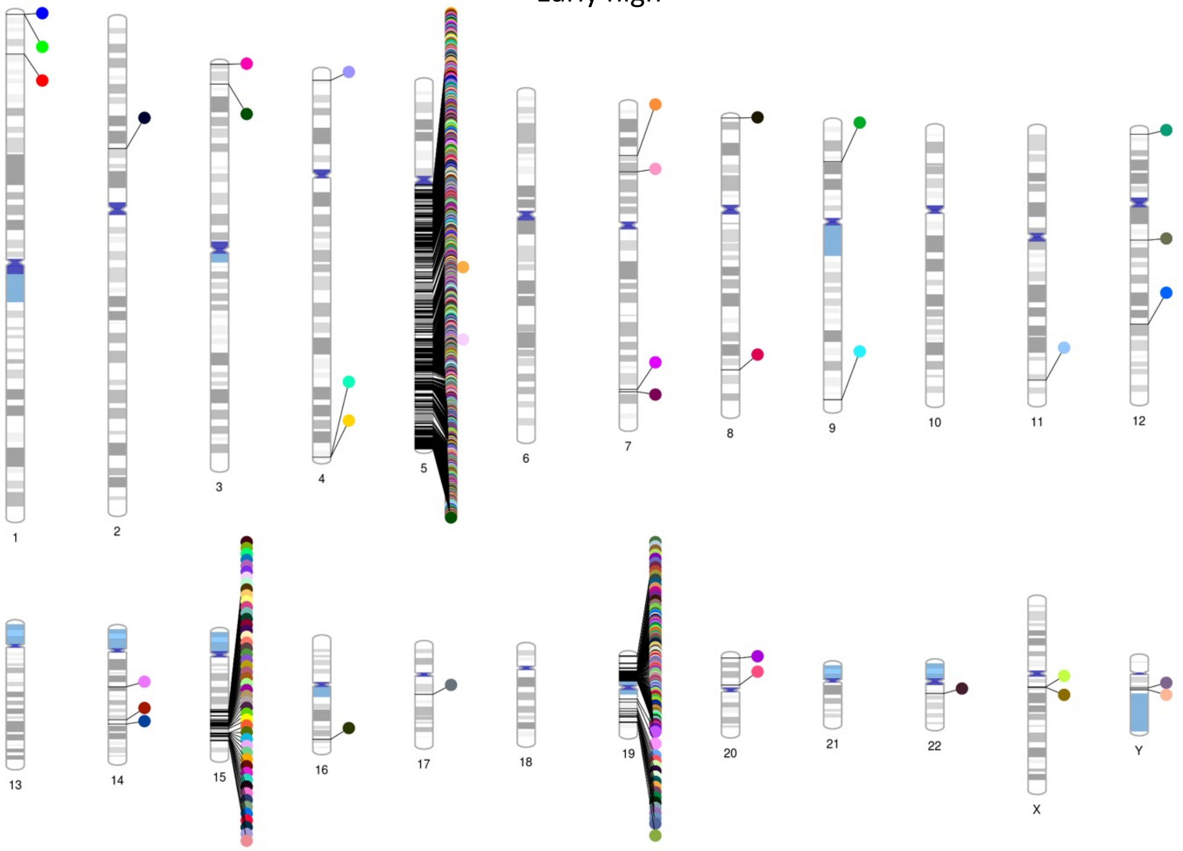

Ashoti et al. Figure S4

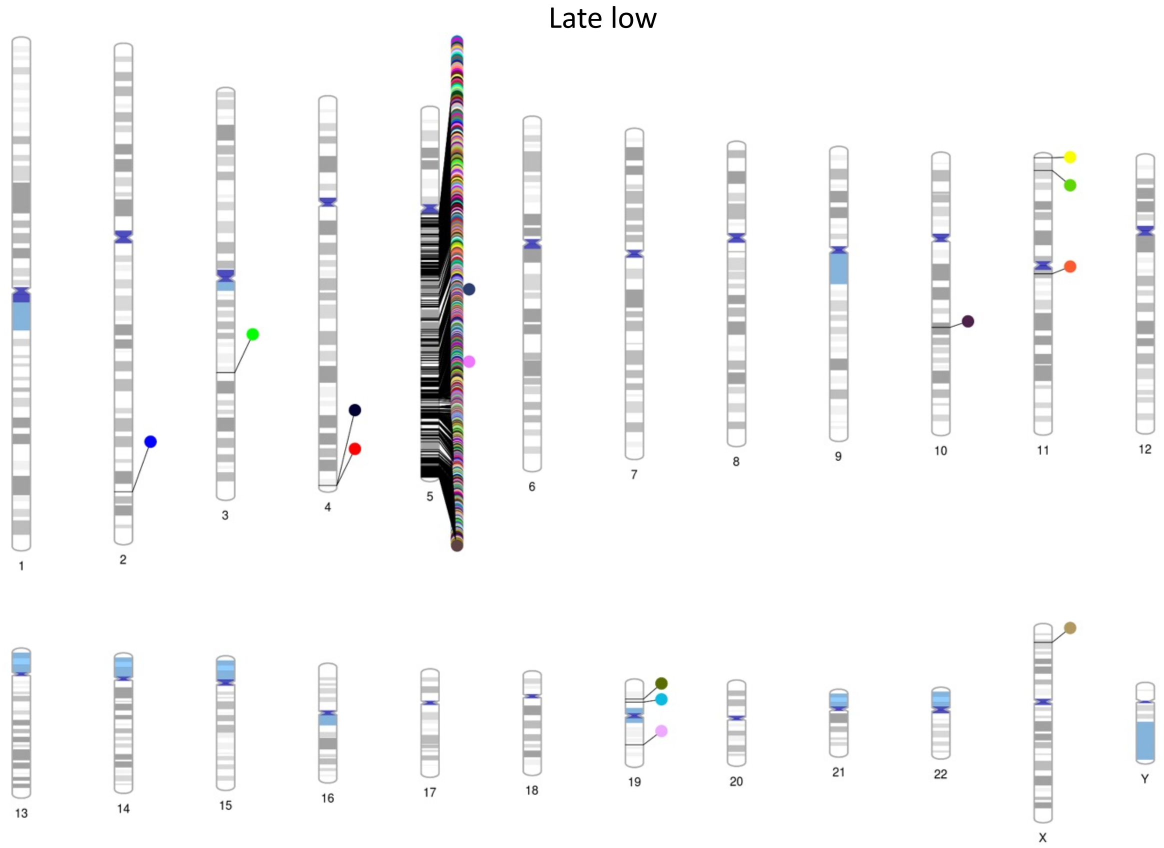

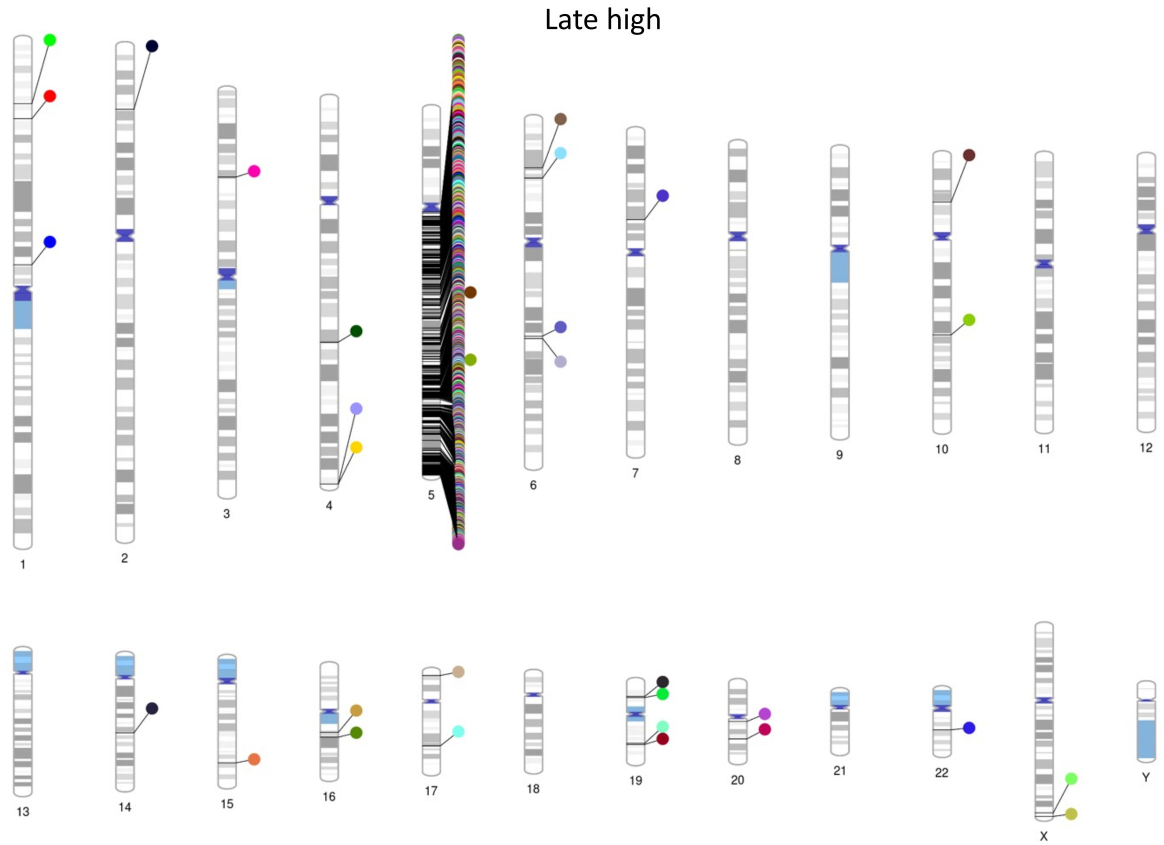

Ashoti et al. Figure S4
