# Supplementary material for: Considerations and practical implications of performing a phenotypic CRISPR/Cas survival screen
Source: PLoS One. 2022 Feb 17;17(2):e0263262. doi: 10.1371/journal.pone.0263262 (PMC8853573; doi:10.1371/journal.pone.0263262)
Supplement: S1 File — (DOCX) [file pone.0263262.s016.docx]

**Supplementary material**

Screen results shown here do not corroborate previous findings of Shadle et al [66] and Lek at al. [67]. S8 Fig shows data plots that display the enrichment (Log2(fold change)) and significance (-Log10(P-value)) of DUX4 and 3 other genes that were initially considered hits. Genes were only considered if a minimum of one sgRNA showed significant enrichment in at least 3 out of 4 screens. Genes involved in the pathways identified by Shadle et al. and Lek et al. did not meet these criteria (S7B and S8A Figs). Furthermore, knocking out these genes in the DIE cells did not show an increased survival compared to background noise (S7 and S8 Figs), as is noticeable in some of the false positives identified during this CRIPSR screen (S7 Fig).

The siRNA screen differs in many aspects to the performed genome-wide CRIPSR/Cas9 screen executed here. The siRNA screen was knocking-down the druggable genome, using Lipofectamine RNAiMAX to deliver the siRNA library, in Rhabdomyosarcoma-derived cells; whereas our screen was knocking out protein-coding genes genome-wide, using a viral library, in chronic myeloid leukemia-derived cells. These differences could explain why results between the two screens are not correlating with one another. Furthermore, A side-by-side comparison study of CRISPR/Cas9 and a next-generation RNAi screen reveals that the screening methods seem to effect different biological aspect of the cells, therefore finding little correlations between results. The authors also in part attribute these differences to the technical differences between the two techniques [86].
