# Supplementary material for: Considerations and practical implications of performing a phenotypic CRISPR/Cas survival screen
Source: PLoS One. 2022 Feb 17;17(2):e0263262. doi: 10.1371/journal.pone.0263262 (PMC8853573; doi:10.1371/journal.pone.0263262)
Supplement: Attachment — Submitted filename: Rebuttal reviewers PloS ONE_Final.docx [file pone.0263262.s017.docx]

Reviewer #1: The manuscript by Ashoti et al. describes a CRISPR/Cas9 rescue screen for modulators of DUX4 toxicity in adherent leukemic cells stably transduced with a lentiviral vector carrying a doxycycline-inducible DUX4 transgene. Unfortunately, the study did not find target genes that could mitigate DUX4 toxicity. The article is easy to understand and well written. I have several comments

* This kind of approach is not new: 1 group has already published a similar approach in muscle cells (Lek et al, Science Translational medicine 2020); 1 group performed a siRNA screen in RD cells (Shadle et al, Plos Genetivs 2017). Lek identified hypoxia signalling inhibitors that attenuate DUX4-induced cell death and Shadle identified MYC-mediated apoptotic pathway and components of the double-stranded RNA (dsRNA) innate immune response. None of these pathways were not found in the current study. This can be related to the cellular system and/or method used. It would be useful to compare the sensitivity to death of myoblasts, adherent leukemic cells and RD cells. Does a same level of DUX4 leads to similar cell death? This could impact the interpretation of the results.

- *We thank the reviewer for the thoughtful and encouraging comments on our manuscript. We compared DUX4 expression between the different manuscripts and observed that the inducible cell model in the Shadle et al. and the Lek et al. screens (see figure below) showed approximately a 100-fold increase in DUX4 expression when comparing induced (4h/8h) to uninduced RD cells (see figure below). Our qPCR data show between 45-89-fold difference in DUX4 expression between 8h-induced DIE cells and uninduced DIE cells. Thus, the induction of DUX4 expression falls approximately in the same range between the different manuscripts. This suggests that indeed a same level of DUX4 leads to similar death in the different cell systems used.*

Shadle et al. 2017. Supp. Figure 5

- *As the reviewer suggested, some of the differences in findings can indeed be explained by the differences in cellular systems that were used in the different manuscripts. For example, myoblasts (used in Lek et al.) respond differently to hypoxia, compared to tumor/cancer cells that we used that have adapted to thrive even through hypoxic conditions.*
- *With respect to the question whether DUX4 expression leads to similar death: one explanation for the differences between the manuscripts is that DUX4-induced apoptosis can occur in multiple ways, and that inhibiting hypoxia-induced apoptosis might not always rescue DUX4-affected cells.*

* The differentially expressed genes have been compared with 4 other studies. Did the authors also compare their results with the Lek study? It may help to find muscle specific targets.

- *We have compared our result with the Lek study. The Lek study found 18 enriched hits. When overlapping these hits with our data, only MED25 was shared as significantly enriched in all our datasets. MED16 and MED24 also showed some enrichment, but most guides did not reach statistical significance (LFC > 1, -log10(p-value) >2) in most of the screens. SIN3B also showed a small but significant enrichment in the early-screen datasets, with 3 out of the 4 guides. However, this gene lies on chromosome 19p, to which the DUX4 transgene has also integrated, and was therefore filtered out of our analysis. Thus, while we have indications that we identified muscle-specific genes, they did not reach statistical significance, which was true for other genes as well.*

* Fig1: the chronology for the different experiments is not clearly indicated. From E, it seems that all the cells are dead, but if so, how to nalyse the genes downstream of DUX4?

- *We have changed the order of the graphs as well as the figure legends to clarify the chronology and the methods of the different experiments.*
- *The cells in figure 1E were treated with doxycycline for at least 24hours. We clarified this in the text. This exposure allowed us to see surviving DUX4 knockout cells when cells were treated with Cas9 and DUX4 sgRNA. However, to analyze downstream DUX4 targets, we used a time frame of 4.5-8.5 hours of doxycycline treatment. This allows us to see the early events that are triggered upon doxycycline induction.*

* Fig1F: A two-tailed student t-test is not the right test to perform when more than 2 populations are compared.

*-We always only compared 2 populations. We clarified this in the figure.*

* qPCR analysis: Are the MIQE guidelines followed? Which statistical analysis was performed to choose HPRT as the normalizer?

- *We followed the MIQE guidelines and added additional information to the Material and methods section, to clarify the procedure in more detail, and to comply as much as possible to the MIQE guidelines. We chose HPRT (after testing 3 different loading controls), after verifying that HPRT did not change more than 2 Ct values between all the different samples (so between treated and untreated). Thus, doxy exposure does not significantly affect HPRT.*

* The authors removed false-positive hits that resulted from Cas9-mediated elimination of either the DUX4 or the rtTA transgenes on chromosomes 5q and 19p. However, they did not remove the false positive hits on chromosome 15, which correlates to the region that has integrated on chromosome 19p. Why?

- *We did not remove these hits as they are related to the MED25 gene, and not the transgene. While these hits turned out to be a false positive, they did not obscure the rest of the data. We also corrected a typo in the text: chromosome 19p should be 19q.*

Minor:
* L186: DUX4 is not described as an “activating” factor.

- *We have adjusted this in the text.*

* A drawing of the generation of the DIE cells, including the final cassettes, might be useful.

- *We have added an updated figure 1A that includes the different constructs that were used and included also the final DUX4 cassette, that allows doxycycline-inducible DUX4 expression.*

* According to NCBI primer blast website, the forward primer for PRAMF1 contains 1 mutation.

- *We thank the reviewer for his sharp eye. The sequence of the primers for PRAMEF1 come from a previously published paper: * Ferreboeuf, M. et al. DUX4 and DUX4 downstream target genes are expressed in fetal FSHD muscles. Hum. Mol. Genet.* ***23****, 171–181 (2014)*
- *The “mutation” is at position 17 of the primer, so at the 5’ end of the primer. The first 16 base pairs are complementary, and the qPCR raw data showed a clear melting curve with only one significant peak. We are therefore confident that the primers yielded the correct PCR product.*

- *Also, the primer sequence was derived from a paper from Ferreboeuf et al. from 2014. The sequence could be a SNV (which would be a missense mutation), which is also indicated as a possibility in ENSEMBL.*

<https://www.ensembl.org/Homo_sapiens/Variation/Mappings?db=core;g=ENSG00000116721;r=1:12791397-12796628;t=ENST00000332296;v=rs1063776;vdb=variation;vf=803761#ENST00000332296_803761_C_tablePanel>

* L174: PAX5 is not described to be linked to FSHD. Only PAX7 was. PAX5 was described to be subject to allele-specific regulation during B-lymphopoiesis (Nutt,

1999).

- *Indeed, PAX5 is expressed in blood cells and is important in B-cell development. Upon comparing gene expression profiles similar to that induced by DUX4, we found a correlation with our DUX4-induced genes and a study that identified PAX5-induced genes. This can be explained by the fact that both DUX4 and PAX genes are homeodomain factors and both bind to homeodomain-binding motifs.*

* The paragraph about the Shadle article should be in the discussion along with a paragraph on the Lek paper.

- *We added information to this paragraph that included and discussed the Lek paper.*

Reviewer #2: Title: A CRISPR/Cas survival screen reveals unexpected mechanisms of rescue

By Ashoti, A. et al.

Ashoti, A. et al. performed an exhaustive genome-wide CRISPR/Cas9 phenotypic rescue screen to identify modulators of cytotoxicity induced by DUX4, a transcription factor that causes Facioscapulohumeral muscular dystrophy (FSHD). Ashoti, A. et al. was not able to find key effectors other than DUX4.

Major comments:

Ashoti, A. et al. aim was to find downstream effectors of DUX4-induced activation of gene expression for the purpose to find potential therapeutic targets or treatment options. By using a doxycycline-inducible cell line, Ashoti, A. et al. was not able to find effectors other than DUX4. Their take home message was to emphasize on the importance of the chosen cell line and CRISPR/Cas9 gRNA library when performing a CRISPR/Cas9 loss-of-function screens. Overall, the paper was well written and does not raise major concerns.

>We thank the reviewer for these comments.

Minor comments:

1) The abstract has some redundant sentences. Also, please write FSHD in full with FSHD abbreviation between brackets. In lines 35-36, the authors put a strong statement about FSHD onset. In some FSHD patients, muscle weakness starts elsewhere.

- *We have changed the wording in the text to make the statement less strong: “As the name implies, FSHD generally starts in the muscles of the face and shoulder girdle”.*

2) Some additional clarifications need to be addressed in figure legends to allow the reader to understand the data without going back to the main text (e.g. state doxycycline concentration, time points, and statistics such as number of replicates etc…).

- *We have added clarifications in the figures legends that allows the reader to understand them without having to go back to the main text.*

3) Line 51: Please find another synonym to manipulability.

- *We have changed it to “adaptability”*
  4) Line 64: “disorderS” in plural.
- *We have adjusted this.*
  5) Lines 86-90: The author has to be careful in its statement about the similarity in the network of DUX4-activated genes between cell lines. For example, the network of DUX4-activated genes in mice is not similar to that found in human cell lines.
- *We have changed the text to clarify this. “The network of genes activated by pioneer factors is therefore less affected by cellular identity, in the human context.”*
- 6) Lines 123-124: The author stated that the sorted cell line as robust cell death upon doxycycline addition without providing any data related to that.
- *We have added FACS data using Annexin5 staining that quantified the portion of DIE cells that stained positive for this marker upon doxycycline induction: 24h after doxycycline treatment only 0.5% of the DIE cells survived compared to 91% of untreated cells. We have added this information to Suppl. Figure 2.*

7) Line 201: The author chose two doxycycline concentrations to perform the screen. However, the author hasn’t provided any evidence about their cytotoxicity? Is there any information that can be provided to show that these chosen concentrations are not toxic to the used cell line?

*Our data demonstrate that the toxicity of doxycycline is mediated by DUX4 expression and not by toxicity of doxycycline. We have shown this in a number of ways, using a high concentration of doxycline (1000nl/ml), which was the highest concentration in the screens. We observed the following:*

1. *We have shown that doxycycline (1000ng/ml) does not induce cell death in the DIE cell line when DUX4 was knocked out (DIE-KO) (Fig. 1E).*
2. *When comparing gene expression in untreated versus doxycycline (1000ng/ml)-treated DIE cells or DIE-KO cells, there was a robust induction of gene expression in treated DIE cells compared to untreated cells but not in treated DIE-KO cells compared to untreated cells.*
3. *We have examined cell death in the parental cell line treated with doxycycline (1000ng/ml), treated or untreated DIE cells by live cell imaging and observed a very robust induction of cell death in treated DIE cells but not in untreated DIE cells or the treated parental cell line. The small induction of cell death in untreated DIE cells or the treated parental cell line can be attributed to the suboptimal conditions at the microscope.*

*Altogether, these data show that with the highest doxycycline concentration there is little toxic effect on the cells.*

8) Lines 221-22: Please be consistent in the “rtTA3” nomenclature.

- *We have adjusted this in the figures and in the text.*

9) Line 300: “virtual” is not a written English.

- *We have changed this to “virtual”.*

10) Line 312: Please put “of” between “…as well as levels” and DUX4 expression.

- *We have adjusted this.*
  11) Line 317: Please remove “its” from the sentence.
- *We have removed ‘its’.*

12) Line 331: Please replace “effect” with affect.

- *We have replaced it.*

13) Line 339: “Despite our library only TARGETS protein-coding genes…

- *We have changed it to “While sgRNAs in our library only target protein-coding genes …”*

14) Line 340: “…picked up any mitigating non-coding gRNAs…”. –

- *We have changed it to RNAs.*

15) Line 430: “BsmBI sites with directly adjacent TO the tracrRNA sequence…”.

- We have adjusted this.
  16) Line 441: “…extracted by addition OF chloroform…”.
- *We have adjusted this.*

17) Line 479 and 483: 600 thousands reads should be written with a comma not at dot.

- *We have adjusted this.*

18) Line 532: 6-7

- *We have adjusted this to 6-7 days.*

19) Figure 2E: The colors of the different dots are indistinguishable. Please chose better representation of the data.

- *We have changed the colors and have also made every sample a different bullet point, to better represent the data.*

20) Figure 4E: the authors measured number of eGFP positive cells. Measuring eGFP fluorescent intensity is more accurate as a representation of knock-out efficiency.

- *We have clarified the figure to indicate that on the Y-axis the GFP intensity is shown.*

Reviewer #3: This is an important study demonstrating DUX4 is the only key effector for FSHD disease. It was shown that no single gene knockout other than DUX4 could rescue DUX4-triggered apoptosis in their transgene model. The study is well done, and the paper is nicely prepared. There are few comments;
Minore;
1- in line 29, "...identify essential genes or genes that are lethal." could this sentence be re-written to prevent redundancy in genes and genes words? or could the "essential genes" be deleted and only use "...genes..." in this sentence?

- *We have adjusted this to remove the redundant part.*

2- Authored mentioned that only DUX4 knockout can rescue DUX4-triggered apoptosis in their in vitro system. They used CRISPR-Cas9 gRNA to knockout the DUX4 gene, but we know that DUX4 and other DUX genes in the human genome have very similar sequences. Do authors think that their gRNA may target all DUX genes, including DUXA, DUXC, etc., along with DUX4? If true, Do authors believe that it is difficult to say that only DUX4 knockout is essential for preventing apoptosis? Please explain more in this case.

- *Since we have overexpressed DUX4 and other DUX genes are not expressed (except for the DUX4 transgene), there will not be an effect of the gRNA targeting DUX4.*
- *We introduced DUX4 as a doxycycline-inducible transgene, and upon doxycycline admission the cells died. This cell death is rescued with 6 different DUX4-specific guides.*
- *The sgRNAs were designed so that they would specifically target the gene that they were designed for. Given the little overlap in sequence between DUX4 and the other DUX genes, the chance of a guide RNA that targets DUX4 to target other DUX genes is negligible.*

3- Authors used 24 and 72 hours as early and late time points for doxycycline treatment (204-205). Since DUX4 is so toxic and its expression in patient biopsies is very low which can not be detected easily by western blot or qRT-PCR, do author think that timepoints below 24hr may show lower cell a different pattern of gene expression they found in 24hr and 72hr more relevant to FSHD patient ones?

- *We agree with the reviewer that time points shorter than 24h will show gene expression that is more relevant to FSHD patients. In a system where DUX4 expression is inducible, these shorter time points will show DUX4-induced expression and provides insight into the network of genes that is induced downstream of DUX4. Given this, we analyzed gene expression in DIE cells 4.5h or 8.5h after doxycycline induction (Fig. 2A).*
- *In contrast, in our screen we allowed the induced DIE cells to recover after doxycycline exposure for 24h or 72h respectively. This allowed amplification of the surviving cells, and subsequent identification of the guide RNA in the surviving cells. At earlier time points, we would have taken along a lot of cells that are in the process of dying. This would give too much background.*

4- Recently published paper shows that DUX4 mRNA silencing using U7-snRNA can prevent apoptosis in FSHD patient myoblasts and myotubes and reduce DUX4-activated biomarkers (DOI:<https://doi.org/10.1016/j.omtn.2020.12.004>). Authors can compare their results with this study's results since both demonstrate the importance of DUX4 silencing as a disease modulator.

- *Similar to this paper, we also show that abrogating DUX4 expression prevents apoptosis in our cell line, underscoring the importance of DUX4 as a therapeutic target. In our paper we focused on identifying DUX4 targets that account for its toxic effect, with the hope to find potential targets in addition to DUX4. We clarified the importance of DUX4 silencing as a disease modulator in the manuscript.*
