# Supplementary material for: Considerations and practical implications of performing a phenotypic CRISPR/Cas survival screen
Source: PLoS One. 2022 Feb 17;17(2):e0263262. doi: 10.1371/journal.pone.0263262 (PMC8853573; doi:10.1371/journal.pone.0263262)
Supplement: Attachment — Submitted filename: Rebuttal letter revision.docx [file pone.0263262.s018.docx]

**Rebuttal letter revision**

In Fig. 1F, the student t-test cannot be used as the Reviewer 1 correctly pointed out.

>In Fig. 1D as well as in Fig. 1F, we have re-analyzed our data using ANOVA analysis (using Prism software). We have indicated the significance in our graphs for the relevant comparisons and can provide the ANOVA analysis if needed.

While we acknowledge errors in the prior analysis, the raw data unequivocally prove induction of doxycycline-mediated DUX4 expression in DIE cells, as well as DUX4-mediated gene expression. We have now plotted the data as fold change (2^-ddCt) upon doxycycline treatment. Upon comparing doxycycline-mediated induction of DUX4 or DUX4 target genes, only doxycycline treatment in DIE cells yielded significant induction of these genes. We also included different doses of doxycyline demonstrating dose-dependent induction of DUX4 expression in DIE cells.

In addition, as the Reviewer 1 pointed out, the MIQE guideline (PMID: 19246619) states in “8.1 normalization” that “Normalization against a single reference gene is not acceptable unless the investigators present clear evidence for the reviewers that confirms its invariant expression under the experimental conditions described. The optimal number and choice of reference genes must be experimentally determined and the method reported (PMIDs: 12184808; 11972351; 15289330).” Please follow their guidelines to show your reference genes are suitable for normalization. I was also not convinced by your argument about Ct values.

>We agree with the reviewer that clear evidence should be presented for the invariant expressioni of reference genes. We included (1) data showing that doxycycline treatment does not influence gene expression in DIE cells, using three reference genes (HPRT, 18S and GAPDH) and (2) data showing the invariant expression of HPRT under the described experimental conditions (Suppl. Fig. S2). We have also included these data below.

Please submit your revised manuscript by Jan 13 2022 11:59PM. If you will need more time than this to complete your revisions, please reply to this message or contact the journal office at [plosone@plos.org](mailto:plosone@plos.org). When you're ready to submit your revision, log on to https://www.editorialmanager.com/pone/ and select the 'Submissions Needing Revision' folder to locate your manuscript file.

Please include the following items when submitting your revised manuscript:

- A rebuttal letter that responds to each point raised by the academic editor and reviewer(s). You should upload this letter as a separate file labeled 'Response to Reviewers'.
- A marked-up copy of your manuscript that highlights changes made to the original version. You should upload this as a separate file labeled 'Revised Manuscript with Track Changes'.
- An unmarked version of your revised paper without tracked changes. You should upload this as a separate file labeled 'Manuscript'.

If you would like to make changes to your financial disclosure, please include your updated statement in your cover letter. Guidelines for resubmitting your figure files are available below the reviewer comments at the end of this letter.

If applicable, we recommend that you deposit your laboratory protocols in protocols.io to enhance the reproducibility of your results. Protocols.io assigns your protocol its own identifier (DOI) so that it can be cited independently in the future. For instructions see: <https://journals.plos.org/plosone/s/submission-guidelines#loc-laboratory-protocols>. Additionally, PLOS ONE offers an option for publishing peer-reviewed Lab Protocol articles, which describe protocols hosted on protocols.io. Read more information on sharing protocols at <https://plos.org/protocols?utm_medium=editorial-email&utm_source=authorletters&utm_campaign=protocols>.

We look forward to receiving your revised manuscript.

Kind regards,

Hodaka Fujii, M.D., Ph.D.
Academic Editor
PLOS ONE

Journal Requirements:

Please review your reference list to ensure that it is complete and correct. If you have cited papers that have been retracted, please include the rationale for doing so in the manuscript text, or remove these references and replace them with relevant current references. Any changes to the reference list should be mentioned in the rebuttal letter that accompanies your revised manuscript. If you need to cite a retracted article, indicate the article’s retracted status in the References list and also include a citation and full reference for the retraction notice.

[Note: HTML markup is below. Please do not edit.]

Reviewers' comments:

Reviewer's Responses to Questions

**Comments to the Author**

1. If the authors have adequately addressed your comments raised in a previous round of review and you feel that this manuscript is now acceptable for publication, you may indicate that here to bypass the “Comments to the Author” section, enter your conflict of interest statement in the “Confidential to Editor” section, and submit your "Accept" recommendation.

Reviewer #1: All comments have been addressed

Reviewer #3: All comments have been addressed

2. Is the manuscript technically sound, and do the data support the conclusions?

The manuscript must describe a technically sound piece of scientific research with data that supports the conclusions. Experiments must have been conducted rigorously, with appropriate controls, replication, and sample sizes. The conclusions must be drawn appropriately based on the data presented.

Reviewer #1: Yes

Reviewer #3: Yes

3. Has the statistical analysis been performed appropriately and rigorously?

Reviewer #1: No

Reviewer #3: Yes

4. Have the authors made all data underlying the findings in their manuscript fully available?

The [PLOS Data policy](http://www.plosone.org/static/policies.action#sharing) requires authors to make all data underlying the findings described in their manuscript fully available without restriction, with rare exception (please refer to the Data Availability Statement in the manuscript PDF file). The data should be provided as part of the manuscript or its supporting information, or deposited to a public repository. For example, in addition to summary statistics, the data points behind means, medians and variance measures should be available. If there are restrictions on publicly sharing data—e.g. participant privacy or use of data from a third party—those must be specified.

Reviewer #1: Yes

Reviewer #3: Yes

5. Is the manuscript presented in an intelligible fashion and written in standard English?

PLOS ONE does not copyedit accepted manuscripts, so the language in submitted articles must be clear, correct, and unambiguous. Any typographical or grammatical errors should be corrected at revision, so please note any specific errors here.

Reviewer #1: Yes

Reviewer #3: Yes

6. Review Comments to the Author

Please use the space provided to explain your answers to the questions above. You may also include additional comments for the author, including concerns about dual publication, research ethics, or publication ethics. (Please upload your review as an attachment if it exceeds 20,000 characters)

Reviewer #1: Overall, the authors responded to questions and comments. However, I do not approve of the way the reference gene was chosen for the qPCR (no statistical analysis but instead they verify that the reference gene did not change more than 2 Ct values between all the different samples). Why 2 Ct values? how do you know if 2Ct is acceptable? an ANOVA analysis should have been performed.
For fig.1F, I previously mentioned that A two-tailed student t-test is not the right test to perform when more than 2
populations are compared. The authors answered that they always only compared 2 populations. But again, this is not rigorous when you have several populations on the same graph. Again, a one-way ANOVA should have been used and not a T-test.

Reviewer #3: This is a very well written paper and authors appropriately addressed all reviewer's comments. The manuscript is acceptable to be published.

7. PLOS authors have the option to publish the peer review history of their article ([what does this mean?](https://journals.plos.org/plosone/s/editorial-and-peer-review-process#loc-peer-review-history)). If published, this will include your full peer review and any attached files.


If you choose “no”, your identity will remain anonymous but your review may still be made public.


**Do you want your identity to be public for this peer review?** For information about this choice, including consent withdrawal, please see our [Privacy Policy](https://www.plos.org/privacy-policy).

Reviewer #1: No

Reviewer #3: No
