# Supplementary material for: Considerations and practical implications of performing a phenotypic CRISPR/Cas survival screen
Source: PLoS One. 2022 Feb 17;17(2):e0263262. doi: 10.1371/journal.pone.0263262 (PMC8853573; doi:10.1371/journal.pone.0263262)
Supplement: Peer review history

# Peer review: Decision Letter 0

**DOI:** 10.1371/journal.pone.0263262.r001

**Authors:** Hodaka Fujii

28 Jun 2021

PONE-D-21-15750

A CRISPR/Cas survival screen reveals unexpected mechanisms of rescue

PLOS ONE

Dear Dr. Geijsen,

Thank you for submitting your manuscript to PLOS ONE. After careful consideration, we feel that it has merit but does not fully meet PLOS ONE’s publication criteria as it currently stands. Therefore, we invite you to submit a revised version of the manuscript that addresses the points raised during the review process.

Your manuscript has been reviewed by three experts. Please revise it according to their suggestions.

Please submit your revised manuscript by Aug 08 2021 11:59PM. If you will need more time than this to complete your revisions, please reply to this message or contact the journal office at plosone@plos.org. When you're ready to submit your revision, log on to https://www.editorialmanager.com/pone/ and select the 'Submissions Needing Revision' folder to locate your manuscript file.

Please include the following items when submitting your revised manuscript:

- A rebuttal letter that responds to each point raised by the academic editor and reviewer(s). You should upload this letter as a separate file labeled 'Response to Reviewers'.
- A marked-up copy of your manuscript that highlights changes made to the original version. You should upload this as a separate file labeled 'Revised Manuscript with Track Changes'.
- An unmarked version of your revised paper without tracked changes. You should upload this as a separate file labeled 'Manuscript'.

If you would like to make changes to your financial disclosure, please include your updated statement in your cover letter. Guidelines for resubmitting your figure files are available below the reviewer comments at the end of this letter.

If applicable, we recommend that you deposit your laboratory protocols in protocols.io to enhance the reproducibility of your results. Protocols.io assigns your protocol its own identifier (DOI) so that it can be cited independently in the future. For instructions see: http://journals.plos.org/plosone/s/submission-guidelines#loc-laboratory-protocols. Additionally, PLOS ONE offers an option for publishing peer-reviewed Lab Protocol articles, which describe protocols hosted on protocols.io. Read more information on sharing protocols at https://plos.org/protocols?utm_medium=editorial-email&utm_source=authorletters&utm_campaign=protocols.

We look forward to receiving your revised manuscript.

Kind regards,

Hodaka Fujii, M.D., Ph.D.

Academic Editor

PLOS ONE

Journal Requirements:

Please review your reference list to ensure that it is complete and correct. If you have cited papers that have been retracted, please include the rationale for doing so in the manuscript text, or remove these references and replace them with relevant current references. Any changes to the reference list should be mentioned in the rebuttal letter that accompanies your revised manuscript. If you need to cite a retracted article, indicate the article’s retracted status in the References list and also include a citation and full reference for the retraction notice.

When submitting your revision, we need you to address these additional requirements.

1. Please ensure that your manuscript meets PLOS ONE's style requirements, including those for file naming. The PLOS ONE style templates can be found at

https://journals.plos.org/plosone/s/file?id=wjVg/PLOSOne_formatting_sample_main_body.pdf and

https://journals.plos.org/plosone/s/file?id=ba62/PLOSOne_formatting_sample_title_authors_affiliations.pdf

2. In your Methods section, please provide additional details regarding the cell lines used in your study and ensure you have described the source. For more information regarding PLOS' policy on materials sharing and reporting, see https://journals.plos.org/plosone/s/materials-and-software-sharing#loc-sharing-materials, and for more information on PLOS ONE's guidelines for research using cell lines, see https://journals.plos.org/plosone/s/submission-guidelines#loc-cell-lines.

[Note: HTML markup is below. Please do not edit.]

Reviewers' comments:

Reviewer's Responses to Questions

**Comments to the Author**

1. Is the manuscript technically sound, and do the data support the conclusions?

The manuscript must describe a technically sound piece of scientific research with data that supports the conclusions. Experiments must have been conducted rigorously, with appropriate controls, replication, and sample sizes. The conclusions must be drawn appropriately based on the data presented. 

Reviewer #1: Yes

Reviewer #2: Yes

Reviewer #3: Yes

**********

2. Has the statistical analysis been performed appropriately and rigorously? 

Reviewer #1: Yes

Reviewer #2: Yes

Reviewer #3: Yes

**********

3. Have the authors made all data underlying the findings in their manuscript fully available?

The PLOS Data policy requires authors to make all data underlying the findings described in their manuscript fully available without restriction, with rare exception (please refer to the Data Availability Statement in the manuscript PDF file). The data should be provided as part of the manuscript or its supporting information, or deposited to a public repository. For example, in addition to summary statistics, the data points behind means, medians and variance measures should be available. If there are restrictions on publicly sharing data—e.g. participant privacy or use of data from a third party—those must be specified.

Reviewer #1: Yes

Reviewer #2: Yes

Reviewer #3: Yes

**********

4. Is the manuscript presented in an intelligible fashion and written in standard English?

PLOS ONE does not copyedit accepted manuscripts, so the language in submitted articles must be clear, correct, and unambiguous. Any typographical or grammatical errors should be corrected at revision, so please note any specific errors here.

Reviewer #1: Yes

Reviewer #2: Yes

Reviewer #3: Yes

**********

5. Review Comments to the Author

Please use the space provided to explain your answers to the questions above. You may also include additional comments for the author, including concerns about dual publication, research ethics, or publication ethics. (Please upload your review as an attachment if it exceeds 20,000 characters)

Reviewer #1: The manuscript by Ashoti et al. describes a CRISPR/Cas9 rescue screen for modulators of DUX4 toxicity in adherent leukemic cells stably transduced with a lentiviral vector carrying a doxycycline-inducible DUX4 transgene. Unfortunately, the study did not find target genes that could mitigate DUX4 toxicity. The article is easy to understand and well written. I have several comments

This kind of approach is not new: 1 group has already published a similar approach in muscle cells (Lek et al, Science Translational medicine 2020); 1 group performed a siRNA screen in RD cells (Shadle et al, Plos Genetivs 2017). Lek identified hypoxia signalling inhibitors that attenuate DUX4-induced cell death and Shadle identified MYC-mediated apoptotic pathway and components of the double-stranded RNA (dsRNA) innate immune response. None of these pathways were not found in the current study. This can be related to the cellular system and/or method used. It would be useful to compare the sensitivity to death of myoblasts, adherent leukemic cells and RD cells. Does a same level of DUX4 leads to similar cell death? This could impact the interpretation of the results.

The differentially expressed genes have been compared with 4 other studies. Did the authors also compare their results with the Lek study? It may help to find muscle specific targets.

Fig1: the chronology for the different experiments is not clearly indicated. From E, it seems that all the cells are dead, but if so, how to nalyse the genes downstream of DUX4?

Fig1F: A two-tailed student t-test is not the right test to perform when more than 2 populations are compared

qPCR analysis: Are the MIQE guidelines followed? Which statistical analysis was performed to choose HPRT as the normalizer?

The authors removed false-positive hits that resulted from Cas9-mediated elimination of either the DUX4 or the rtTA transgenes on chromosomes 5q and 19p. However, they did not remove the false positive hits on chromosome 15, which correlates to the region that has integrated on chromosome 19p. Why?

Minor:

L186: DUX4 is not described as an “activating” factor.

A drawing of the generation of the DIE cells, including the final cassettes, might be useful.

According to NCBI primer blast website, the forward primer for PRAMF1 contains 1 mutation.

L174: PAX5 is not described to be linked to FSHD. Only PAX7 was. PAX5 was described to be subject to allele-specific regulation during B-lymphopoiesis (Nutt, 1999).

The paragraph about the Shadle article should be in the discussion along with a paragraph on the Lek paper.

Reviewer #2: Title: A CRISPR/Cas survival screen reveals unexpected mechanisms of rescue

By Ashoti, A. et al.

Ashoti, A. et al. performed an exhaustive genome-wide CRISPR/Cas9 phenotypic rescue screen to identify modulators of cytotoxicity induced by DUX4, a transcription factor that causes Facioscapulohumeral muscular dystrophy (FSHD). Ashoti, A. et al. was not able to find key effectors other than DUX4.

Major comments:

Ashoti, A. et al. aim was to find downstream effectors of DUX4-induced activation of gene expression for the purpose to find potential therapeutic targets or treatment options. By using a doxycycline-inducible cell line, Ashoti, A. et al. was not able to find effectors other than DUX4. Their take home message was to emphasize on the importance of the chosen cell line and CRISPR/Cas9 gRNA library when performing a CRISPR/Cas9 loss-of-function screens. Overall, the paper was well written and does not raise major concerns.

Minor comments:

1) The abstract has some redundant sentences. Also, please write FSHD in full with FSHD abbreviation between brackets. In lines 35-36, the authors put a strong statement about FSHD onset. In some FSHD patients, muscle weakness starts elsewhere.

2) Some additional clarifications need to be addressed in figure legends to allow the reader to understand the data without going back to the main text (e.g. state doxycycline concentration, time points, and statistics such as number of replicates etc…).

3) Line 51: Please find another synonym to manipulability.

4) Line 64: “disorderS” in plural.

5) Lines 86-90: The author has to be careful in its statement about the similarity in the network of DUX4-activated genes between cell lines. For example, the network of DUX4-activated genes in mice is not similar to that found in human cell lines.

6) Lines 123-124: The author stated that the sorted cell line as robust cell death upon doxycycline addition without providing any data related to that.

7) Line 201: The author chose two doxycycline concentrations to perform the screen. However, the author hasn’t provided any evidence about their cytotoxicity? Is there any information that can be provided to show that these chosen concentrations are not toxic to the used cell line?

8) Lines 221-22: Please be consistent in the “rtTA3” nomenclature.

9) Line 300: “virtual” is not a written English.

10) Line 312: Please put “of” between “…as well as levels” and DUX4 expression.

11) Line 317: Please remove “its” from the sentence.

12) Line 331: Please replace “effect” with affect.

13) Line 339: “Despite our library only TARGETS protein-coding genes…

14) Line 340: “…picked up any mitigating non-coding gRNAs…”.

15) Line 430: “BsmBI sites with directly adjacent TO the tracrRNA sequence…”.

16) Line 441: “…extracted by addition OF chloroform…”.

17) Line 479 and 483: 600 thousands reads should be written with a comma not at dot.

18) Line 532: is it 6-7 days?

19) Figure 2E: The colors of the different dots are indistinguishable. Please chose better representation of the data.

20) Figure 4E: the authors measured number of eGFP positive cells. Measuring eGFP fluorescent intensity is more accurate as a representation of knock-out efficiency.

Reviewer #3: This is an important study demonstrating DUX4 is the only key effector for FSHD disease. It was shown that no single gene knockout other than DUX4 could rescue DUX4-triggered apoptosis in their transgene model. The study is well done, and the paper is nicely prepared. There are few comments;

Minore;

1- in line 29, "...identify essential genes or genes that are lethal." could this sentence be re-written to prevent redundancy in genes and genes words? or could the "essential genes" be deleted and only use "...genes..." in this sentence?

2- Authored mentioned that only DUX4 knockout can rescue DUX4-triggered apoptosis in their in vitro system. They used CRISPR-Cas9 gRNA to knockout the DUX4 gene, but we know that DUX4 and other DUX genes in the human genome have very similar sequences. Do authors think that their gRNA may target all DUX genes, including DUXA, DUXC, etc., along with DUX4? If true, Do authors believe that it is difficult to say that only DUX4 knockout is essential for preventing apoptosis? Please explain more in this case.

3- Authors used 24 and 72 hours as early and late time points for doxycycline treatment (204-205). Since DUX4 is so toxic and its expression in patient biopsies is very low which can not be detected easily by western blot or qRT-PCR, do author think that timepoints below 24hr may show lower cell a different pattern of gene expression they found in 24hr and 72hr more relevant to FSHD patient ones?

4- Recently published paper shows that DUX4 mRNA silencing using U7-snRNA can prevent apoptosis in FSHD patient myoblasts and myotubes and reduce DUX4-activated biomarkers (DOI:https://doi.org/10.1016/j.omtn.2020.12.004). Authors can compare their results with this study's results since both demonstrate the importance of DUX4 silencing as a disease modulator.

**********

6. PLOS authors have the option to publish the peer review history of their article (what does this mean?). If published, this will include your full peer review and any attached files.

If you choose “no”, your identity will remain anonymous but your review may still be made public.

**Do you want your identity to be public for this peer review?** For information about this choice, including consent withdrawal, please see our Privacy Policy.

Reviewer #1: No

Reviewer #2: No

Reviewer #3: No

[NOTE: If reviewer comments were submitted as an attachment file, they will be attached to this email and accessible via the submission site. Please log into your account, locate the manuscript record, and check for the action link "View Attachments". If this link does not appear, there are no attachment files.]

While revising your submission, please upload your figure files to the Preflight Analysis and Conversion Engine (PACE) digital diagnostic tool, https://pacev2.apexcovantage.com/. PACE helps ensure that figures meet PLOS requirements. To use PACE, you must first register as a user. Registration is free. Then, login and navigate to the UPLOAD tab, where you will find detailed instructions on how to use the tool. If you encounter any issues or have any questions when using PACE, please email PLOS at figures@plos.org. Please note that Supporting Information files do not need this step.

# Author comment: Author response to Decision Letter 0

**DOI:** 10.1371/journal.pone.0263262.r002

25 Oct 2021

Reviewer #1: The manuscript by Ashoti et al. describes a CRISPR/Cas9 rescue screen for modulators of DUX4 toxicity in adherent leukemic cells stably transduced with a lentiviral vector carrying a doxycycline-inducible DUX4 transgene. Unfortunately, the study did not find target genes that could mitigate DUX4 toxicity. The article is easy to understand and well written. I have several comments

* This kind of approach is not new: 1 group has already published a similar approach in muscle cells (Lek et al, Science Translational medicine 2020); 1 group performed a siRNA screen in RD cells (Shadle et al, Plos Genetivs 2017). Lek identified hypoxia signalling inhibitors that attenuate DUX4-induced cell death and Shadle identified MYC-mediated apoptotic pathway and components of the double-stranded RNA (dsRNA) innate immune response. None of these pathways were not found in the current study. This can be related to the cellular system and/or method used. It would be useful to compare the sensitivity to death of myoblasts, adherent leukemic cells and RD cells. Does a same level of DUX4 leads to similar cell death? This could impact the interpretation of the results.

- We thank the reviewer for the thoughtful and encouraging comments on our manuscript. We compared DUX4 expression between the different manuscripts and observed that the inducible cell model in the Shadle et al. and the Lek et al. screens (see figure below) showed approximately a 100-fold increase in DUX4 expression when comparing induced (4h/8h) to uninduced RD cells (see figure below). Our qPCR data show between 45-89-fold difference in DUX4 expression between 8h-induced DIE cells and uninduced DIE cells. Thus, the induction of DUX4 expression falls approximately in the same range between the different manuscripts. This suggests that indeed a same level of DUX4 leads to similar death in the different cell systems used.

Shadle et al. 2017. Supp. Figure 5

- As the reviewer suggested, some of the differences in findings can indeed be explained by the differences in cellular systems that were used in the different manuscripts. For example, myoblasts (used in Lek et al.) respond differently to hypoxia, compared to tumor/cancer cells that we used that have adapted to thrive even through hypoxic conditions. 

- With respect to the question whether DUX4 expression leads to similar death: one explanation for the differences between the manuscripts is that DUX4-induced apoptosis can occur in multiple ways, and that inhibiting hypoxia-induced apoptosis might not always rescue DUX4-affected cells. 

* The differentially expressed genes have been compared with 4 other studies. Did the authors also compare their results with the Lek study? It may help to find muscle specific targets.

- We have compared our result with the Lek study. The Lek study found 18 enriched hits. When overlapping these hits with our data, only MED25 was shared as significantly enriched in all our datasets. MED16 and MED24 also showed some enrichment, but most guides did not reach statistical significance (LFC > 1, -log10(p-value) >2) in most of the screens. SIN3B also showed a small but significant enrichment in the early-screen datasets, with 3 out of the 4 guides. However, this gene lies on chromosome 19p, to which the DUX4 transgene has also integrated, and was therefore filtered out of our analysis. Thus, while we have indications that we identified muscle-specific genes, they did not reach statistical significance, which was true for other genes as well. 

* Fig1: the chronology for the different experiments is not clearly indicated. From E, it seems that all the cells are dead, but if so, how to nalyse the genes downstream of DUX4?

- We have changed the order of the graphs as well as the figure legends to clarify the chronology and the methods of the different experiments.

- The cells in figure 1E were treated with doxycycline for at least 24hours. We clarified this in the text. This exposure allowed us to see surviving DUX4 knockout cells when cells were treated with Cas9 and DUX4 sgRNA. However, to analyze downstream DUX4 targets, we used a time frame of 4.5-8.5 hours of doxycycline treatment. This allows us to see the early events that are triggered upon doxycycline induction.

* Fig1F: A two-tailed student t-test is not the right test to perform when more than 2 populations are compared. 

-We always only compared 2 populations. We clarified this in the figure.

* qPCR analysis: Are the MIQE guidelines followed? Which statistical analysis was performed to choose HPRT as the normalizer?

- We followed the MIQE guidelines and added additional information to the Material and methods section, to clarify the procedure in more detail, and to comply as much as possible to the MIQE guidelines. We chose HPRT (after testing 3 different loading controls), after verifying that HPRT did not change more than 2 Ct values between all the different samples (so between treated and untreated). Thus, doxy exposure does not significantly affect HPRT. 

* The authors removed false-positive hits that resulted from Cas9-mediated elimination of either the DUX4 or the rtTA transgenes on chromosomes 5q and 19p. However, they did not remove the false positive hits on chromosome 15, which correlates to the region that has integrated on chromosome 19p. Why?

- We did not remove these hits as they are related to the MED25 gene, and not the transgene. While these hits turned out to be a false positive, they did not obscure the rest of the data. We also corrected a typo in the text: chromosome 19p should be 19q. 

Minor:

* L186: DUX4 is not described as an “activating” factor.

- We have adjusted this in the text.

* A drawing of the generation of the DIE cells, including the final cassettes, might be useful.

- We have added an updated figure 1A that includes the different constructs that were used and included also the final DUX4 cassette, that allows doxycycline-inducible DUX4 expression.

* According to NCBI primer blast website, the forward primer for PRAMF1 contains 1 mutation.

- We thank the reviewer for his sharp eye. The sequence of the primers for PRAMEF1 come from a previously published paper: * Ferreboeuf, M. et al. DUX4 and DUX4 downstream target genes are expressed in fetal FSHD muscles. Hum. Mol. Genet. 23, 171–181 (2014)

- The “mutation” is at position 17 of the primer, so at the 5’ end of the primer. The first 16 base pairs are complementary, and the qPCR raw data showed a clear melting curve with only one significant peak. We are therefore confident that the primers yielded the correct PCR product. 

- Also, the primer sequence was derived from a paper from Ferreboeuf et al. from 2014. The sequence could be a SNV (which would be a missense mutation), which is also indicated as a possibility in ENSEMBL. 

https://www.ensembl.org/Homo_sapiens/Variation/Mappings?db=core;g=ENSG00000116721;r=1:12791397-12796628;t=ENST00000332296;v=rs1063776;vdb=variation;vf=803761#ENST00000332296_803761_C_tablePanel

* L174: PAX5 is not described to be linked to FSHD. Only PAX7 was. PAX5 was described to be subject to allele-specific regulation during B-lymphopoiesis (Nutt, 

1999).

- Indeed, PAX5 is expressed in blood cells and is important in B-cell development. Upon comparing gene expression profiles similar to that induced by DUX4, we found a correlation with our DUX4-induced genes and a study that identified PAX5-induced genes. This can be explained by the fact that both DUX4 and PAX genes are homeodomain factors and both bind to homeodomain-binding motifs.

* The paragraph about the Shadle article should be in the discussion along with a paragraph on the Lek paper.

- We added information to this paragraph that included and discussed the Lek paper.

Reviewer #2: Title: A CRISPR/Cas survival screen reveals unexpected mechanisms of rescue

By Ashoti, A. et al.

Ashoti, A. et al. performed an exhaustive genome-wide CRISPR/Cas9 phenotypic rescue screen to identify modulators of cytotoxicity induced by DUX4, a transcription factor that causes Facioscapulohumeral muscular dystrophy (FSHD). Ashoti, A. et al. was not able to find key effectors other than DUX4.

Major comments:

Ashoti, A. et al. aim was to find downstream effectors of DUX4-induced activation of gene expression for the purpose to find potential therapeutic targets or treatment options. By using a doxycycline-inducible cell line, Ashoti, A. et al. was not able to find effectors other than DUX4. Their take home message was to emphasize on the importance of the chosen cell line and CRISPR/Cas9 gRNA library when performing a CRISPR/Cas9 loss-of-function screens. Overall, the paper was well written and does not raise major concerns.

>We thank the reviewer for these comments.

Minor comments:

1) The abstract has some redundant sentences. Also, please write FSHD in full with FSHD abbreviation between brackets. In lines 35-36, the authors put a strong statement about FSHD onset. In some FSHD patients, muscle weakness starts elsewhere.

- We have changed the wording in the text to make the statement less strong: “As the name implies, FSHD generally starts in the muscles of the face and shoulder girdle”.

2) Some additional clarifications need to be addressed in figure legends to allow the reader to understand the data without going back to the main text (e.g. state doxycycline concentration, time points, and statistics such as number of replicates etc…).

- We have added clarifications in the figures legends that allows the reader to understand them without having to go back to the main text.

3) Line 51: Please find another synonym to manipulability.

- We have changed it to “adaptability”

4) Line 64: “disorderS” in plural. 

- We have adjusted this.

5) Lines 86-90: The author has to be careful in its statement about the similarity in the network of DUX4-activated genes between cell lines. For example, the network of DUX4-activated genes in mice is not similar to that found in human cell lines.

- We have changed the text to clarify this. “The network of genes activated by pioneer factors is therefore less affected by cellular identity, in the human context.” 

- 

6) Lines 123-124: The author stated that the sorted cell line as robust cell death upon doxycycline addition without providing any data related to that.

- We have added FACS data using Annexin5 staining that quantified the portion of DIE cells that stained positive for this marker upon doxycycline induction: 24h after doxycycline treatment only 0.5% of the DIE cells survived compared to 91% of untreated cells. We have added this information to Suppl. Figure 2.

7) Line 201: The author chose two doxycycline concentrations to perform the screen. However, the author hasn’t provided any evidence about their cytotoxicity? Is there any information that can be provided to show that these chosen concentrations are not toxic to the used cell line?

Our data demonstrate that the toxicity of doxycycline is mediated by DUX4 expression and not by toxicity of doxycycline. We have shown this in a number of ways, using a high concentration of doxycline (1000nl/ml), which was the highest concentration in the screens. We observed the following:

1. We have shown that doxycycline (1000ng/ml) does not induce cell death in the DIE cell line when DUX4 was knocked out (DIE-KO) (Fig. 1E).

2. When comparing gene expression in untreated versus doxycycline (1000ng/ml)-treated DIE cells or DIE-KO cells, there was a robust induction of gene expression in treated DIE cells compared to untreated cells but not in treated DIE-KO cells compared to untreated cells.

3. We have examined cell death in the parental cell line treated with doxycycline (1000ng/ml), treated or untreated DIE cells by live cell imaging and observed a very robust induction of cell death in treated DIE cells but not in untreated DIE cells or the treated parental cell line. The small induction of cell death in untreated DIE cells or the treated parental cell line can be attributed to the suboptimal conditions at the microscope. 

Altogether, these data show that with the highest doxycycline concentration there is little toxic effect on the cells. 

8) Lines 221-22: Please be consistent in the “rtTA3” nomenclature. 

- We have adjusted this in the figures and in the text.

9) Line 300: “virtual” is not a written English.

- We have changed this to “virtual”.

10) Line 312: Please put “of” between “…as well as levels” and DUX4 expression.

- We have adjusted this.

11) Line 317: Please remove “its” from the sentence.

- We have removed ‘its’.

12) Line 331: Please replace “effect” with affect.

- We have replaced it.

13) Line 339: “Despite our library only TARGETS protein-coding genes…

- We have changed it to “While sgRNAs in our library only target protein-coding genes …”

14) Line 340: “…picked up any mitigating non-coding gRNAs…”. –

- We have changed it to RNAs.

15) Line 430: “BsmBI sites with directly adjacent TO the tracrRNA sequence…”.

- We have adjusted this.

16) Line 441: “…extracted by addition OF chloroform…”.

- We have adjusted this.

17) Line 479 and 483: 600 thousands reads should be written with a comma not at dot.

- We have adjusted this.

18) Line 532: 6-7 

- We have adjusted this to 6-7 days.

19) Figure 2E: The colors of the different dots are indistinguishable. Please chose better representation of the data.

- We have changed the colors and have also made every sample a different bullet point, to better represent the data. 

20) Figure 4E: the authors measured number of eGFP positive cells. Measuring eGFP fluorescent intensity is more accurate as a representation of knock-out efficiency.

- We have clarified the figure to indicate that on the Y-axis the GFP intensity is shown. 

Reviewer #3: This is an important study demonstrating DUX4 is the only key effector for FSHD disease. It was shown that no single gene knockout other than DUX4 could rescue DUX4-triggered apoptosis in their transgene model. The study is well done, and the paper is nicely prepared. There are few comments;

Minore;

1- in line 29, "...identify essential genes or genes that are lethal." could this sentence be re-written to prevent redundancy in genes and genes words? or could the "essential genes" be deleted and only use "...genes..." in this sentence? 

- We have adjusted this to remove the redundant part.

2- Authored mentioned that only DUX4 knockout can rescue DUX4-triggered apoptosis in their in vitro system. They used CRISPR-Cas9 gRNA to knockout the DUX4 gene, but we know that DUX4 and other DUX genes in the human genome have very similar sequences. Do authors think that their gRNA may target all DUX genes, including DUXA, DUXC, etc., along with DUX4? If true, Do authors believe that it is difficult to say that only DUX4 knockout is essential for preventing apoptosis? Please explain more in this case.

- Since we have overexpressed DUX4 and other DUX genes are not expressed (except for the DUX4 transgene), there will not be an effect of the gRNA targeting DUX4.

- We introduced DUX4 as a doxycycline-inducible transgene, and upon doxycycline admission the cells died. This cell death is rescued with 6 different DUX4-specific guides.

- The sgRNAs were designed so that they would specifically target the gene that they were designed for. Given the little overlap in sequence between DUX4 and the other DUX genes, the chance of a guide RNA that targets DUX4 to target other DUX genes is negligible.

3- Authors used 24 and 72 hours as early and late time points for doxycycline treatment (204-205). Since DUX4 is so toxic and its expression in patient biopsies is very low which can not be detected easily by western blot or qRT-PCR, do author think that timepoints below 24hr may show lower cell a different pattern of gene expression they found in 24hr and 72hr more relevant to FSHD patient ones?

- We agree with the reviewer that time points shorter than 24h will show gene expression that is more relevant to FSHD patients. In a system where DUX4 expression is inducible, these shorter time points will show DUX4-induced expression and provides insight into the network of genes that is induced downstream of DUX4. Given this, we analyzed gene expression in DIE cells 4.5h or 8.5h after doxycycline induction (Fig. 2A). 

- In contrast, in our screen we allowed the induced DIE cells to recover after doxycycline exposure for 24h or 72h respectively. This allowed amplification of the surviving cells, and subsequent identification of the guide RNA in the surviving cells. At earlier time points, we would have taken along a lot of cells that are in the process of dying. This would give too much background. 

4- Recently published paper shows that DUX4 mRNA silencing using U7-snRNA can prevent apoptosis in FSHD patient myoblasts and myotubes and reduce DUX4-activated biomarkers (DOI:https://doi.org/10.1016/j.omtn.2020.12.004). Authors can compare their results with this study's results since both demonstrate the importance of DUX4 silencing as a disease modulator.

- Similar to this paper, we also show that abrogating DUX4 expression prevents apoptosis in our cell line, underscoring the importance of DUX4 as a therapeutic target. In our paper we focused on identifying DUX4 targets that account for its toxic effect, with the hope to find potential targets in addition to DUX4. We clarified the importance of DUX4 silencing as a disease modulator in the manuscript.

Attachment Submitted filename: Rebuttal reviewers PloS ONE_Final.docx Click here for additional data file.

# Peer review: Decision Letter 1

**DOI:** 10.1371/journal.pone.0263262.r003

**Authors:** Hodaka Fujii

29 Nov 2021

PONE-D-21-15750R1Considerations and practical implications of performing a phenotypic CRISPR/Cas survival screen

PLOS ONE

Dear Dr. Geijsen,

Thank you for submitting your manuscript to PLOS ONE. After careful consideration, we feel that it has merit but does not fully meet PLOS ONE’s publication criteria as it currently stands. Therefore, we invite you to submit a revised version of the manuscript that addresses the points raised during the review process.

In Fig. 1F, the student t-test cannot be used as the Reviewer 1 correctly pointed out. This is because you were implicitly comparing 6 samples and performed multiple t-tests. In such cases, “Even if all null hypotheses were true, the chance that at least one of these P values would be less than 0.05 by coincidence is far higher than 5%.” (from “Intuitive Biostatistics”, Oxford University Press, pp. 255). In fact, this is the most common error in scientific publications. Please use ANOVA as  the Reviewer 1 suggested. Also, please read a textbook of statistics such as one mentioned above and get assistance from statisticians about this issue.

In addition, as the Reviewer 1 pointed out, the MIQE guideline (PMID: 19246619) states in “8.1 normalization” that “Normalization against a single reference gene is not acceptable unless the investigators present clear evidence for the reviewers that confirms its invariant expression under the experimental conditions described. The optimal number and choice of reference genes must be experimentally determined and the method reported (PMIDs: 12184808; 11972351; 15289330).” Please follow their guidelines to show your reference genes are suitable for normalization. I was also not convinced by your argument about Ct values.

Please submit your revised manuscript by Jan 13 2022 11:59PM. If you will need more time than this to complete your revisions, please reply to this message or contact the journal office at plosone@plos.org. When you're ready to submit your revision, log on to https://www.editorialmanager.com/pone/ and select the 'Submissions Needing Revision' folder to locate your manuscript file.

Please include the following items when submitting your revised manuscript:

- A rebuttal letter that responds to each point raised by the academic editor and reviewer(s). You should upload this letter as a separate file labeled 'Response to Reviewers'.
- A marked-up copy of your manuscript that highlights changes made to the original version. You should upload this as a separate file labeled 'Revised Manuscript with Track Changes'.
- An unmarked version of your revised paper without tracked changes. You should upload this as a separate file labeled 'Manuscript'.

If you would like to make changes to your financial disclosure, please include your updated statement in your cover letter. Guidelines for resubmitting your figure files are available below the reviewer comments at the end of this letter.

If applicable, we recommend that you deposit your laboratory protocols in protocols.io to enhance the reproducibility of your results. Protocols.io assigns your protocol its own identifier (DOI) so that it can be cited independently in the future. For instructions see: https://journals.plos.org/plosone/s/submission-guidelines#loc-laboratory-protocols. Additionally, PLOS ONE offers an option for publishing peer-reviewed Lab Protocol articles, which describe protocols hosted on protocols.io. Read more information on sharing protocols at https://plos.org/protocols?utm_medium=editorial-email&utm_source=authorletters&utm_campaign=protocols.

We look forward to receiving your revised manuscript.

Kind regards,

Hodaka Fujii, M.D., Ph.D.

Academic Editor

PLOS ONE

Journal Requirements:

Please review your reference list to ensure that it is complete and correct. If you have cited papers that have been retracted, please include the rationale for doing so in the manuscript text, or remove these references and replace them with relevant current references. Any changes to the reference list should be mentioned in the rebuttal letter that accompanies your revised manuscript. If you need to cite a retracted article, indicate the article’s retracted status in the References list and also include a citation and full reference for the retraction notice.

[Note: HTML markup is below. Please do not edit.]

Reviewers' comments:

Reviewer's Responses to Questions

**Comments to the Author**

1. If the authors have adequately addressed your comments raised in a previous round of review and you feel that this manuscript is now acceptable for publication, you may indicate that here to bypass the “Comments to the Author” section, enter your conflict of interest statement in the “Confidential to Editor” section, and submit your "Accept" recommendation.

Reviewer #1: All comments have been addressed

Reviewer #3: All comments have been addressed

**********

2. Is the manuscript technically sound, and do the data support the conclusions?

The manuscript must describe a technically sound piece of scientific research with data that supports the conclusions. Experiments must have been conducted rigorously, with appropriate controls, replication, and sample sizes. The conclusions must be drawn appropriately based on the data presented. 

Reviewer #1: Yes

Reviewer #3: Yes

**********

3. Has the statistical analysis been performed appropriately and rigorously? 

Reviewer #1: No

Reviewer #3: Yes

**********

4. Have the authors made all data underlying the findings in their manuscript fully available?

The PLOS Data policy requires authors to make all data underlying the findings described in their manuscript fully available without restriction, with rare exception (please refer to the Data Availability Statement in the manuscript PDF file). The data should be provided as part of the manuscript or its supporting information, or deposited to a public repository. For example, in addition to summary statistics, the data points behind means, medians and variance measures should be available. If there are restrictions on publicly sharing data—e.g. participant privacy or use of data from a third party—those must be specified.

Reviewer #1: Yes

Reviewer #3: Yes

**********

5. Is the manuscript presented in an intelligible fashion and written in standard English?

PLOS ONE does not copyedit accepted manuscripts, so the language in submitted articles must be clear, correct, and unambiguous. Any typographical or grammatical errors should be corrected at revision, so please note any specific errors here.

Reviewer #1: Yes

Reviewer #3: Yes

**********

6. Review Comments to the Author

Please use the space provided to explain your answers to the questions above. You may also include additional comments for the author, including concerns about dual publication, research ethics, or publication ethics. (Please upload your review as an attachment if it exceeds 20,000 characters)

Reviewer #1: Overall, the authors responded to questions and comments. However, I do not approve of the way the reference gene was chosen for the qPCR (no statistical analysis but instead they verify that the reference gene did not change more than 2 Ct values between all the different samples). Why 2 Ct values? how do you know if 2Ct is acceptable? an ANOVA analysis should have been performed.

For fig.1F, I previously mentioned that A two-tailed student t-test is not the right test to perform when more than 2

populations are compared. The authors answered that they always only compared 2 populations. But again, this is not rigorous when you have several populations on the same graph. Again, a one-way ANOVA should have been used and not a T-test.

Reviewer #3: This is a very well written paper and authors appropriately addressed all reviewer's comments. The manuscript is acceptable to be published.

**********

7. PLOS authors have the option to publish the peer review history of their article (what does this mean?). If published, this will include your full peer review and any attached files.

If you choose “no”, your identity will remain anonymous but your review may still be made public.

**Do you want your identity to be public for this peer review?** For information about this choice, including consent withdrawal, please see our Privacy Policy.

Reviewer #1: No

Reviewer #3: No

[NOTE: If reviewer comments were submitted as an attachment file, they will be attached to this email and accessible via the submission site. Please log into your account, locate the manuscript record, and check for the action link "View Attachments". If this link does not appear, there are no attachment files.]

While revising your submission, please upload your figure files to the Preflight Analysis and Conversion Engine (PACE) digital diagnostic tool, https://pacev2.apexcovantage.com/. PACE helps ensure that figures meet PLOS requirements. To use PACE, you must first register as a user. Registration is free. Then, login and navigate to the UPLOAD tab, where you will find detailed instructions on how to use the tool. If you encounter any issues or have any questions when using PACE, please email PLOS at figures@plos.org. Please note that Supporting Information files do not need this step.

# Author comment: Author response to Decision Letter 1

**DOI:** 10.1371/journal.pone.0263262.r004

24 Dec 2021

Rebuttal letter revision

In Fig. 1F, the student t-test cannot be used as the Reviewer 1 correctly pointed out. 

>In Fig. 1D as well as in Fig. 1F, we have re-analyzed our data using ANOVA analysis (using Prism software). We have indicated the significance in our graphs for the relevant comparisons and can provide the ANOVA analysis if needed. 

While we acknowledge errors in the prior analysis, the raw data unequivocally prove induction of doxycycline-mediated DUX4 expression in DIE cells, as well as DUX4-mediated gene expression. We have now plotted the data as fold change (2^-ddCt) upon doxycycline treatment. Upon comparing doxycycline-mediated induction of DUX4 or DUX4 target genes, only doxycycline treatment in DIE cells yielded significant induction of these genes. We also included different doses of doxycyline demonstrating dose-dependent induction of DUX4 expression in DIE cells.

In addition, as the Reviewer 1 pointed out, the MIQE guideline (PMID: 19246619) states in “8.1 normalization” that “Normalization against a single reference gene is not acceptable unless the investigators present clear evidence for the reviewers that confirms its invariant expression under the experimental conditions described. The optimal number and choice of reference genes must be experimentally determined and the method reported (PMIDs: 12184808; 11972351; 15289330).” Please follow their guidelines to show your reference genes are suitable for normalization. I was also not convinced by your argument about Ct values.

>We agree with the reviewer that clear evidence should be presented for the invariant expressioni of reference genes. We included (1) data showing that doxycycline treatment does not influence gene expression in DIE cells, using three reference genes (HPRT, 18S and GAPDH) and (2) data showing the invariant expression of HPRT under the described experimental conditions (Suppl. Fig. S2).

Attachment Submitted filename: Rebuttal letter revision.docx Click here for additional data file.

# Peer review: Decision Letter 2

**DOI:** 10.1371/journal.pone.0263262.r005

**Authors:** Hodaka Fujii

18 Jan 2022

Considerations and practical implications of performing a phenotypic CRISPR/Cas survival screen

PONE-D-21-15750R2

Dear Dr. Geijsen,

We’re pleased to inform you that your manuscript has been judged scientifically suitable for publication and will be formally accepted for publication once it meets all outstanding technical requirements.

Within one week, you’ll receive an e-mail detailing the required amendments. When these have been addressed, you’ll receive a formal acceptance letter and your manuscript will be scheduled for publication.

An invoice for payment will follow shortly after the formal acceptance. To ensure an efficient process, please log into Editorial Manager at http://www.editorialmanager.com/pone/, click the 'Update My Information' link at the top of the page, and double check that your user information is up-to-date. If you have any billing related questions, please contact our Author Billing department directly at authorbilling@plos.org.

If your institution or institutions have a press office, please notify them about your upcoming paper to help maximize its impact. If they’ll be preparing press materials, please inform our press team as soon as possible -- no later than 48 hours after receiving the formal acceptance. Your manuscript will remain under strict press embargo until 2 pm Eastern Time on the date of publication. For more information, please contact onepress@plos.org.

Kind regards,

Hodaka Fujii, M.D., Ph.D.

Academic Editor

PLOS ONE

Additional Editor Comments (optional):

Reviewers' comments:

Reviewer's Responses to Questions

**Comments to the Author**

1. If the authors have adequately addressed your comments raised in a previous round of review and you feel that this manuscript is now acceptable for publication, you may indicate that here to bypass the “Comments to the Author” section, enter your conflict of interest statement in the “Confidential to Editor” section, and submit your "Accept" recommendation.

Reviewer #3: All comments have been addressed

**********

2. Is the manuscript technically sound, and do the data support the conclusions?

The manuscript must describe a technically sound piece of scientific research with data that supports the conclusions. Experiments must have been conducted rigorously, with appropriate controls, replication, and sample sizes. The conclusions must be drawn appropriately based on the data presented. 

Reviewer #3: Yes

**********

3. Has the statistical analysis been performed appropriately and rigorously? 

Reviewer #3: Yes

**********

4. Have the authors made all data underlying the findings in their manuscript fully available?

The PLOS Data policy requires authors to make all data underlying the findings described in their manuscript fully available without restriction, with rare exception (please refer to the Data Availability Statement in the manuscript PDF file). The data should be provided as part of the manuscript or its supporting information, or deposited to a public repository. For example, in addition to summary statistics, the data points behind means, medians and variance measures should be available. If there are restrictions on publicly sharing data—e.g. participant privacy or use of data from a third party—those must be specified.

Reviewer #3: Yes

**********

5. Is the manuscript presented in an intelligible fashion and written in standard English?

PLOS ONE does not copyedit accepted manuscripts, so the language in submitted articles must be clear, correct, and unambiguous. Any typographical or grammatical errors should be corrected at revision, so please note any specific errors here.

Reviewer #3: Yes

**********

6. Review Comments to the Author

Please use the space provided to explain your answers to the questions above. You may also include additional comments for the author, including concerns about dual publication, research ethics, or publication ethics. (Please upload your review as an attachment if it exceeds 20,000 characters)

Reviewer #3: This is an important paper and the authors appropriately addressed all comments. The paper is acceptable to be published.

**********

7. PLOS authors have the option to publish the peer review history of their article (what does this mean?). If published, this will include your full peer review and any attached files.

If you choose “no”, your identity will remain anonymous but your review may still be made public.

**Do you want your identity to be public for this peer review?** For information about this choice, including consent withdrawal, please see our Privacy Policy.

Reviewer #3: No

# Editor report: Acceptance letter

**DOI:** 10.1371/journal.pone.0263262.r006

**Authors:** Hodaka Fujii

24 Jan 2022

PONE-D-21-15750R2 

Considerations and practical implications of performing a phenotypic CRISPR/Cas survival screen 

Dear Dr. Geijsen:

I'm pleased to inform you that your manuscript has been deemed suitable for publication in PLOS ONE. Congratulations! Your manuscript is now with our production department. 

If your institution or institutions have a press office, please let them know about your upcoming paper now to help maximize its impact. If they'll be preparing press materials, please inform our press team within the next 48 hours. Your manuscript will remain under strict press embargo until 2 pm Eastern Time on the date of publication. For more information please contact onepress@plos.org.

If we can help with anything else, please email us at plosone@plos.org. 

Thank you for submitting your work to PLOS ONE and supporting open access. 

Kind regards, 

PLOS ONE Editorial Office Staff

on behalf of

Dr. Hodaka Fujii 

Academic Editor

PLOS ONE
